# Explainable Machine Learning Predictive Models for Surgical Site Infections: Scoping Review

**DOI:** 10.2196/94617

**Published:** 2026-09-30

**Authors:** Rui Sun, Yao Liu, Shuya Lu, Ermira Tartari, Gabriel Birgand, Lin Yang, Lei Zhou

**Affiliations:** 1 Public Health Emergency Center, Chinese Center for Disease Control and Prevention, Beijing, China; 2 School of Nursing, The Hong Kong Polytechnic University, 11 Yuk Choi Rd, Hong Kong Special Administrative Region, China (Hong Kong), +85227666398; 3 Faculty of Health Sciences, University of Malta, Msida, Malta; 4 Cibles et Médicaments des Infections et de l' Immunité – UR 1155, Nantes Université, Nantes, France; 5 Centre d' Appui à la Prévention des Infections Associées aux Soins des Pays de la Loire, Centre Hospitalier Universitaire (CHU) - Le Tourville, Nantes, France; 6 National Institute for Health Research Health Protection Research Unit in Healthcare Associated Infections and Antimicrobial Resistance, Imperial College London, London, United Kingdom; 7 Research Centre of Textiles for Future Fashion, The Hong Kong Polytechnic University, Hong Kong Special Administrative Region, China (Hong Kong); 8 National Key Laboratory of Intelligent Tracking and Forecasting for Infectious Diseases, Chinese Center for Disease Control and Prevention, Beijing, China

**Keywords:** surgical site infection, machine learning, explainable artificial intelligence, predictive models, scoping review

## Abstract

**Background:**

Surgical site infections (SSIs) remain a major cause of health care–associated infections, and early prediction is essential for improving patient outcomes. Machine learning (ML) has shown potential for SSI prediction; however, clinical implementation requires models that are both accurate and explainable. Despite recent progress in explainable ML, its clinical application to SSI prediction remains limited.

**Objective:**

This study aimed to map explainable ML models for SSI prediction from a clinical perspective and examine their use of structured and unstructured data across the dimensions of data, methodology, and explanation output.

**Methods:**

We conducted a scoping review following PRISMA-ScR (Preferred Reporting Items for Systematic Reviews and Meta-Analyses extension for Scoping Reviews) and Joanna Briggs Institute (JBI) guidance, and registered the protocol in PROSPERO. Six databases were searched for eligible studies published from January 2010 onward, without language restrictions. The search was conducted on August 9, 2025, and updated on July 14, 2026. We included studies that developed or validated an explainable ML model for predicting SSI in adults. Two reviewers (RS and YL) independently screened studies and extracted data. Findings were narratively synthesized and presented in evidence maps. Methodological quality was assessed using the PROBAST+AI (Prediction model Risk Of Bias Assessment Tool for prediction models using regression or artificial intelligence) methods.

**Results:**

Overall, 77 studies reporting 98 ML models were included. Most models were prognostic (72/98, 73.5%), whereas 26 focused on postoperative SSI diagnosis. A total of 81.8% (63/77) of studies addressed a single surgical specialty, most commonly gastrointestinal surgery (27/63, 42.9%). Overall, 51.9% (40/77) of studies addressed composite SSI predictions. Among all models, % (40/98) were black-box models explained by post hoc methods; SHAP combined with ensemble learning was the leading approach (18/40, 45%). Regression models accounted for half of the inherently interpretable models, interpreted using coefficients. Prognostic models commonly included health and lifestyle (60/72, 83.3%), individual characteristics, and surgical process details (both 57/72, 79.2%); health and lifestyle factors were most frequently important across SSI types. Diagnostic models commonly included surgical process details (13/26, 50%), administrative codes, and individual characteristics (both 10/26, 38.5%). Key diagnostic predictors varied by SSI types: postoperative clinical interventions predominated for composite SSI; vital signs, postoperative interventions, and administrative codes for superficial SSI; postoperative recovery status for deep SSI; and vital signs for organ-space SSI.

**Conclusions:**

Extending previous reviews focusing on model performance, this review mapped explainability methods and important features in SSI prediction, identifying recurring predictor patterns and substantial methodological heterogeneity across prognostic and diagnostic settings. Incomplete reporting of feature definitions and explanatory rationale, together with limited clinical relevance, constrained clinical interpretation and actionability. Clinician-informed reporting frameworks and validation of explanation fidelity and clinical relevance are needed to improve the trustworthiness and utility of SSI prediction models.

## Introduction

Surgical site infection (SSI) is defined as an infection occurring at or near the surgical incision within 30 days after surgery, or within 1 year in the presence of implants [1]. SSIs are classified as superficial, deep, or organ-space infections according to the depth and anatomical location of the affected tissue [2]. These infections can substantially worsen postoperative outcomes by prolonging hospital stay [3], increasing readmission risk [4], and raising mortality [5]. In addition, the need for further treatment imposes a considerable economic burden on both patients and health care systems [6]. Despite major advances in medicine, SSI remains one of the most common health care–associated infections worldwide [7], with reported incidence ranging from 0.5% to 11% [8-12]. This highlights the need for earlier and more comprehensive SSI surveillance [13-16].

As a pivotal subset of AI, machine learning (ML) leverages iterative algorithms to extract intricate patterns from data, ultimately automating clinical prediction [17]. In earlier predictive modeling studies, generalized linear models [18], particularly logistic regression [19], were widely used. However, because disease-related predictors often span multiple domains, traditional regression-based approaches may not adequately capture the complexity of real-world data, especially when relationships are nonlinear [20]. As a result, more complex models, such as deep neural networks and ensemble learning methods, have emerged and often achieve superior performance on high-dimensional data compared with conventional statistical models [21]. Both generalized regression models and nonlinear models can fall within the broader scope of ML [22].

ML models have been increasingly applied to early SSI detection and prediction, including semiautomated or automated surveillance systems [23,24] and risk calculators [25,26]. These models use large and complex health data sources, including electronic health records [27,28], clinical notes [29,30], and thermal images [31,32]. By reducing the need for manual chart review, which is time-consuming and labor-intensive, ML methods may improve the efficiency and accuracy of SSI identification and assessment [33,34] and support more precise clinical decision-making.

Several reviews have shown that ML models can achieve strong and robust performance for SSI detection and prediction. A systematic review reported that ML models for general SSI prediction showed good performance, with a median area under the receiver operating characteristic curve (AUC) of approximately 0.79 in external validation [35]. A meta-analysis found that AI methods for detecting SSI from wound images had excellent discriminative ability and may support automated postdischarge follow-up [36]. Two additional reviews also reported improved performance of ML-based prediction models [37,38].

However, these reviews primarily focused on model development and validation and paid limited attention to how predictions are explained. In clinical practice, clinicians need to understand why a model identifies a patient as high risk to reduce errors and guide appropriate interventions [39-41]. Transparency is therefore essential for improving the clinical validity, trustworthiness, and acceptability of ML-based SSI prediction tools and for supporting their implementation in practice [42].

Explainable methods can improve the transparency of ML models [43]. Under the concept of self-explainability, ML models can be broadly divided into inherently interpretable models and post hoc explainable models. Generalized linear models, decision tree models [44], and Bayesian models [45] are typically considered inherently interpretable because their structures and feature effects are relatively transparent and can be visualized [46]. The ability to understand how these models generate predictions is often referred to as interpretability. By contrast, advanced nonlinear models are usually regarded as black-box models because their internal prediction processes are opaque [47]. To address this limitation without sacrificing predictive performance, post hoc explanation techniques have been developed and are now widely used [46]. When combined with these techniques, black-box models can provide human-readable explanations of their predictions [48]. This is often referred to as explainability. Post hoc explainers include model-specific methods, such as Gini importance for random forests [44], and model-agnostic methods, such as SHAP values [49] and permutation feature importance (PFI) [50]. Together, black-box models and post hoc explainers form post hoc explainable models [46]. However, terminology in this area remains inconsistent, and the terms interpretability and explainability are often used interchangeably [51-53]. To ensure consistency and clarity, this review uses explainability as an umbrella term that includes both explainability and interpretability, consistent with previous studies [52,54].

The explainability approaches described above mainly focus on technical explainability. However, previous studies have emphasized that clinically meaningful explainability should also consider the context of model inputs, the alignment between algorithmic architecture and explanation methods, and the format and interpretive meaning of explanation outputs [55,56]. Using this multidimensional perspective, this scoping review aimed to systematically map how explainability has been implemented and reported in ML-based SSI prediction across three complementary and observable domains: predictors, methods, and outputs. We also sought to identify key evidence and reporting gaps. These evidence maps may provide foundational dimensions and practical entry points for developing a more comprehensive framework to guide the implementation and evaluation of explainability in ML-based SSI prediction.

## Methods

### Protocol Registration

This scoping review was conducted in accordance with the PRISMA-ScR (Preferred Reporting Items for Systematic Reviews and Meta-Analyses extension for Scoping Reviews) guidelines [57] and the Joanna Briggs Institute (JBI) methodological guidance for scoping reviews [58]. The PRISMA (Preferred Reporting Items for Systematic Reviews and Meta-Analyses) checklist is provided in Checklist 1. The protocol was prospectively registered in PROSPERO (CRD420251124760) on September 19, 2025, and subsequently updated on July 14, 2026, to include additional databases and an updated search date. One deviation from the registered protocol occurred: the planned secondary analysis comparing predictor frequency between regression models and black-box ML approaches was not performed because some SSI categories contained too few models for meaningful comparison. All other procedures were conducted as originally specified.

### Information Sources

Reporting of the search strategy followed the PRISMA-S (Preferred Reporting Items for Systematic Reviews and Meta-Analyses literature search extension) [59]. We searched the following electronic databases: MEDLINE (PubMed), Embase, Scopus, Web of Science Core Collection, CINAHL (EBSCOhost), and CNKI. Gray literature was searched separately in Google Scholar (first 200 records screened) and ProQuest Dissertations & Theses Global. Each database was searched independently through its native interface rather than through a single multidatabase platform. Clinical trial registries such as ClinicalTrials.gov did not apply to this study, as this review focused on ML explainability, which is typically described in both the methods and results sections of predictive modeling studies. We restricted the search to studies published from January 2010 onwards to capture the contemporary era of data-driven ML-based infection prediction. No language or other restrictions were applied. The search strategy was developed without validated filters to maximize sensitivity in this emerging field. A comprehensive search was initially run on August 9, 2025, and then rerun on July 14, 2026, using a refined search strategy to identify recent eligible studies. Reference lists of all retrieved reviews were manually screened to identify additional studies not captured by the database search. Conference proceedings identified were reviewed in full text when available. When full texts were unavailable, corresponding authors were contacted by email.

### Search Strategy

An initial limited search of Google Scholar and PubMed was performed to identify relevant records. Text words from titles and abstracts, together with index terms, were used to develop the full search strategy. The final strategy combined controlled vocabulary terms (eg, MeSH terms such as “artificial intelligence,” “machine learning,” and “surgical wound infection”) with free-text terms searched in titles and abstracts (eg, “deep learning,” “neural network*,” and “surgical site infection*”). Terms were organized into 3 concept blocks: AI and machine learning, SSI, and prediction. The PubMed strategy was developed first using MeSH terms and free-text terms, then adapted for Embase (Emtree) and other databases by modifying controlled vocabulary and syntax as appropriate. The initial search strategies of these databases and the subsequent update are provided in Multimedia Appendix 1.

### Eligibility Criteria

Because children and adults differ in risk profiles, exposure patterns, and effect sizes [60-62], this review was restricted to adult populations. The definition of adult was based on the age threshold used in each study’s country. The central concept was the explanation of ML models used to predict SSIs. SSIs included superficial, deep, and organ-space infections according to the criteria of the Centers for Disease Control and Prevention (CDC) and National Healthcare Safety Network (NHSN) [2]. To ensure comprehensive coverage, we also explicitly included specific anatomical variants, such as deep sternal wound infections (DSWI). Other wound types, such as chronic wounds, and other postoperative infectious complications, such as pneumonia and urinary tract infection, were excluded.

We included studies that developed or validated at least one modern explainable ML-based prediction model. ML models were defined as models capable of learning predictive patterns from data through iterative, data-driven training and optimization, including feature selection and hyperparameter tuning [17]. Conventional statistical models primarily used for association testing or hypothesis testing were excluded [63]. All included models had to provide a global explanation using at least one explanatory technique, including both inherently interpretable models and post hoc explainable approaches. Regression-based methods were considered carefully because they may function either as statistical inference tools or as ML prediction models. Following previous work [64], regression models that were knowledge-driven, based on strict assumptions and fixed hyperparameters, were treated as conventional statistical models and excluded. In contrast, regression approaches that incorporated ML characteristics, such as hyperparameter tuning, automated feature selection, or sparsity learning [65], were retained.

We included studies that used structured data or free text to develop predictive models. Studies focused only on nonpredictive tasks or on data types other than structured data or free text were excluded. Purely algorithm-focused studies were also excluded.

### Study Screening and Selection

All retrieved records were imported into EndNote for automated deduplication, after which the remaining records were manually reviewed to identify and remove residual duplicates. Two reviewers (RS and YL) independently screened titles and abstracts to identify potentially relevant studies, followed by full-text screening against the eligibility criteria. Any disagreements were resolved by discussion, and a third reviewer (SYL) was consulted when necessary.

### Data Charting Process

Based on the Checklist for the Critical Appraisal and Data Extraction for Systematic Reviews of Prediction Modelling Studies (CHARMS), a predesigned data charting form was augmented to capture additional predictor and explanation variables specific to machine learning explainability [66]. The form was piloted on the first 10 included studies and revised iteratively. Extracted information included author, year of publication, study objective, setting, participant characteristics (sample size, study period, and surgical procedure), outcome details (classification, definition, and time point of measurement), predictors (source, selection method, collection time window, and model predictors), model development details (model type, algorithm, and performance including discrimination, calibration, and clinical utility), and model explanation details (explainability techniques, visualizations, and outputs).

Because no standardized framework exists for categorizing predictors in SSI prediction models, we developed a structured classification framework based on SSI prevention guidance from the World Health Organization (WHO) and CDC [67,68]. Predictors were organized into three dimensions: patient, surgical, and hospital and contextual factors, across three periods: preoperative, intraoperative, and postoperative. The time point of measurement referred to the reported collection time of candidate predictors.

Models developed using different predictor sets or designed to predict different SSI outcomes were regarded as distinct models. Accordingly, when multiple models developed using the same predictor set or designed to predict the same SSI outcome were reported within a study, only the explainability information from the best-performing model identified in the original study was extracted to balance predictive performance and explainability. Two reviewers (RS and YL) independently extracted the data in Microsoft Excel, and disagreements were resolved by a third reviewer (SYL).

### Synthesis of Results

To summarize the evidence and identify common patterns, we redefined and recategorized model types, predictor categories, and SSI categories. Model type was defined primarily by model purpose. When the original study did not explicitly state the type, two reviewers (RS and YL) classified the model as prognostic or diagnostic based on the temporal relationship between predictor collection and the outcome prediction period, following the operational definition in CHARMS [66] and previous research [69]. Prognostic models estimated the risk of future SSI, whereas diagnostic models assessed the risk of existing SSI. Disagreements were resolved by the third author (SYL).

To better summarize heterogeneous predictors, all candidate predictors were regrouped into clinically meaningful categories based on their definitions and clinical implications, informed by American College of Surgeons National Surgical Quality Improvement Program (ACS-NSQIP) indicator definitions [70] and risk factor taxonomies from previous reviews [13,71,72]. Two reviewers (RS and YL) independently categorized the predictors, and interrater agreement was assessed using Cohen kappa. Disagreements were resolved by the third author (SYL).

Because SSI definitions and risk profiles differ across infection types [73], outcomes were grouped into separate infections and composite infections. Separate infections included superficial, deep, and organ-space infections according to criteria from CDC and NHSN [2]. Composite infections referred to composite SSIs in studies that predicted multiple SSI types within a single ML model.

The review synthesized findings across three dimensions: data-level explainability, methodological explainability, and output-level explainability. An evidence gap map was used to visualize the distribution of evidence across these dimensions. Study characteristics were summarized descriptively and presented in tables. For data-level explainability, the distribution of collection timing and predictor categories across model types was summarized and visualized using bubble matrix plots to assess the clinical interpretability of model inputs.

For methodological explainability, ML models were grouped by algorithm type, and the frequency of explainability techniques was summarized within each algorithm category and displayed in a tree map to show common pairings between ML methods and explainability approaches.

Because ML predictions are generated through learned relationships between input features and outcomes, explainability research often focuses on predictive variables as the central objects of interpretation [74]. Accordingly, output-level explainability focused on the most important features identified across models. For this purpose, the top 10 features in the global feature-importance rankings were extracted and summarized qualitatively across prognostic and diagnostic models and across SSI types using heatmaps, with color intensity reflecting how often each predictor category appeared among the top-ranked features across all models. All visualizations were created using R (version 4.5.2; R Foundation for Statistical Computing). Patterns identified in the evidence map were interpreted narratively to highlight gaps and inform future research priorities.

### Quality and Applicability Assessment

Two reviewers (RS and YL) independently assessed the methodological quality, risk of bias (ROB), and applicability of the included models using the updated PROBAST+AI (Prediction model Risk Of Bias Assessment Tool for prediction models using regression or artificial intelligence) tool [75]. Interrater agreement was evaluated via Cohen kappa (κ), with values interpreted according to Landis and Koch criteria [76]. Disagreements were resolved by a third reviewer (SYL). PROBAST+AI tool comprises 34 signaling questions across two phases: model development (16 items) and validation (18 items). Studies containing both components were evaluated across both phases. For both phases and applicability domains, overall judgments were summarized as low, high, or unclear concern or bias, where low indicates optimal quality and high denotes significant methodological limitations. All analyses were conducted using R version 4.5.2.

## Results

### Study Selection

Figure 1 presents the study selection process. The database search yielded 3365 records. After removal of duplicates and retracted articles, 1972 unique records remained for title and abstract screening, and 1571 were excluded. We assessed 394 full-text articles, of which 75 met the inclusion criteria. An additional 2 studies were identified through citation searching of 19 relevant reviews, resulting in a total of 77 included studies [29,30,39,77-150]. Because some studies developed multiple models with different feature sets or for distinct SSI subtypes, 98 ML models were included in the final analysis.

**Figure 1.**
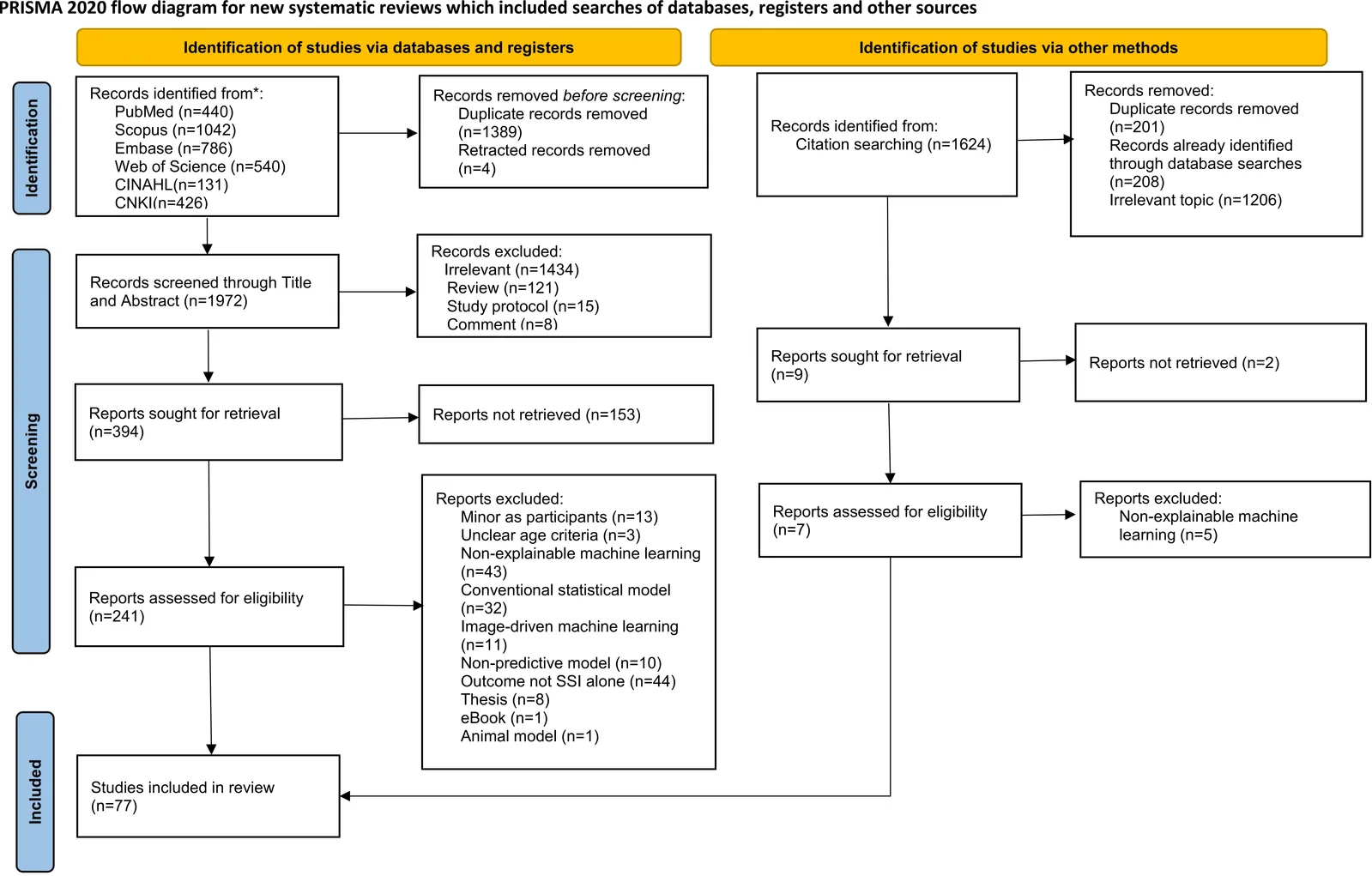
Literature selection process according to the PRISMA (Preferred Reporting Items for Systematic Reviews and Meta-Analyses) guidelines.

### Study and Model Characteristics

The characteristics of the 77 included studies [29,30,39,77-150] and 98 models are summarized in Table 1. The studies were conducted across 14 countries and published between 2014 and 2026, with the majority published after 2021. One study developed both prognostic and diagnostic predictive models [77]. Among the remaining studies, 76.3% developed prognostic models for future SSI risk (58 studies, 71 models) [30,78-82,88-98,105-145], while 18 studies [29,39,83-87,99-104,146-150] developed 25 diagnostic models for detecting existing SSI. Nearly all studies were retrospective, except for 4 prospective cohort studies [78-80,151], and more than half were conducted at a single center. Most studies focused on a single surgical specialty [39,77-80,83,85,87-92,94,96-98,102-109,111-116,119-148,150] (63/77, 81.8%), with gastrointestinal surgery [77-80,83,85,87,94,96,105,108,109,111-115,119-121,126,129,134,139,142,146,148] (27/63, 42.9%) being the most common specialty. The remaining 14 studies [29,30,81,82,84,86,93,95,99-101,117,118,149] developed general models across multiple surgical procedures. A total of 23 studies [78,83,87,88,93,94,104,105,107,108,110,123,130,133,134,137-139,141,143-145,148] did not report their targeted SSI types. Four studies [100,118,119,146] addressed multiple SSI types, whereas the remaining 50 studies [29,30,39,77,79-86,89,90,92,95-99,101-103,106,109,111-115,117,120-122,124-129,131,132,134-136,140,142,147,149,150] aimed to predict a single SSI outcome, including 39 studies [29,30,39,79-86,89,90,92,95-97,101-103,106,109,113-115,117,120,121,124,126,128,129,131,132,135,136,142,149,150] that predicted composite SSI and 11 studies [77,92,98,111,112,122,125,127,134,140,147] that predicted a specific SSI type.

**Table 1.** Evidentiary table of 77 selected publications.

Author and year	Country	Study period	Research site	Target surgery	Predictors source	Target SSI^a^	Model development	Best-performing model
Prognostic prediction (n=59)								
Mamlook et al [117] (2023)	Multiple countries	2013 to 2016	Multiple medical centers	Multiple surgical procedures	ACS-NSQIP^b^ Data	Single (composite SSI^c^)	Seven algorithms	DNN^d^
Van et al [81] (2014)	Multiple countries	2005 to 2010	Multiple medical centers	Multiple surgical procedures	ACS-NSQIP Data	Single (composite SSI)	Seven algorithms	TEXT-SVM^e^
Walczak et al [95](2019)	United States	July to Dec, 2015	A single medical center	Multiple surgical procedures	ACS-NSQIP Data	Single (composite SSI)	Single algorithm	ANN^f^
Bonde et al [118] (2021)	Multiple countries	2012 to 2018	Multiple medical centers	Multiple surgical procedures	ACS-NSQIP Data	Multiple SSIs (including superficial, deep, and organ-space)	Three predictor sets per outcome	DNN
Bonde et al [119] (2024)	United States	2002 to 2018	Multiple medical centers	Gastrointestinal	ACS-NSQIP Data	Multiple SSIs (including superficial, deep, and organ-space)	Two algorithms plus three predictor sets per outcome	DNN
Chen et al [82] (2020)	China	2014 to 2019	A single medical center	Multiple surgical procedures	Institutional EMR^g^	Single (composite SSI)	Six algorithms	The Self-Attention Network
Zhuang et al [30] (2024)	United States	2013 to 2019	Multiple medical centers	Multiple surgical procedures	Institutional EHR^h^	Single (composite SSI)	Single algorithm	LASSO^i^ Regression
Ohno et al [114] (2022)	Japan	2000 to 2018	A single medical center	Gastrointestinal	Institutional EMR	Single (composite SSI^j^)	Single algorithm	An Ensemble Model (Gradient Boosting Tree and Neural Network)
Piebpien et al [109] (2024)	Thailand	2013 to 2019	A single medical center	Gastrointestinal	Hospital and operation databases	Single (composite SSI)	Four algorithms	NB^k^
Yang et al [78] (2024)	China	2021 to 2022	Multiple medical centers	Gastrointestinal	Chinese SSI surveillance cohort	Not reported	Two algorithms	LASSO Regression
Chuyang et al [80] (2017)	Netherlands	2007 to 2009	A single medical center	Gastrointestinal	An open abdominal surgery cohort in the Netherlands	Single (composite SSI) Time to first SSI onset	Three algorithms	An Enhanced Regression Model
Grass et al [120] (2021)	United States	2006 to 2014	A single medical center	Gastrointestinal	ACS-NSQIP data	Single (composite SSI^l^)	Three algorithms	BPMI^m^
Julien et al [79] (2022)	France	2010 to 2017	Multiple medical centers	Gastrointestinal	The REMIND cohort	Single (composite SSI) within 90 days	Single algorithm	RF^n^
Chen et al [121] (2023)	Multiple countries	2012 to 2019	Multiple medical centers	Gastrointestinal	ACS-NSQIP data	Single (composite SSI)	Single algorithm	NN^o^
Isbell et al [111] (2021)	United States	2011 to 2017	A single medical center	Gastrointestinal	Institutional EMR	Single (superficial SSI)	Single algorithm	Regularized Bayesian Multilevel Logistic Regression
Chen et al [113] (2024)	China	2012 to 2022	Multiple medical centers	Gastrointestinal	Institutional EMR	5 perioperative complications (including Composite SSI)	Five algorithms	RF
Salimy et al [122] (2025)	Multiple countries	2013 to 2020	Multiple medical centers	Orthopedics	ACS-NSQIP data	Single (periprosthetic Joint Infection [PJI])	Five algorithms	HGB^p^
Wang et al [123] (2021)	China	2012 to 2019	A single medical center	Orthopedics	Institutional EMR	Not reported	Six algorithms	NB
Wang et al [88] (2025)	China	2018 to 2020	A single medical center	Orthopedics	Institutional EMR	Not reported	Six algorithms	XGBoost^q^
Xiong et al [124] (2022)	China	2019 to 2021	A single medical center	Orthopedics	Institutional EHR; Surgical anesthesia system; Mobile nursing system	Single (composite SSI) within 90 days	Seven algorithms	AdaBoost classification trees
Zhang et al [110] (2024)	China	2015 to 2022	A single medical center	Orthopedics	Institutional EMR	Not reported	Seven algorithms	NB
Golinelli et al [97] (2025)	Italy	2017 to 2021	Multiple medical centers	Orthopedics	Routinely collected health care database	Single (composite SSI)	Four algorithms	XGBoost
Orfanoudaki et al [125] (2022)	Multiple countries	2008 to 2017	Multiple medical centers	Cardiac	STS ACSD^r^ data	5 Surgical outcomes (including deep sternal wound infection)	Five algorithms	XGBoost
Kilic et al [98] (2021)	Multiple countries	2007 to 2017	Multiple medical centers	Cardiac	STS ACSD data	7 Surgical outcomes (including deep sternal wound infection)	Single algorithm	XGBoost
McLean et al [126] (2024)	Multiple countries	2014 and 2016	Multiple medical centers	Gastrointestinal	Prospectively secondary analysis of GlobalSurg-1 cohort and GlobalSurg-2 cohort	Single (composite SSI)	Two algorithms	LASSO logistic regression
Wei et al [112] (2020)	United States	2011 to 2016	A single medical center	Gastrointestinal	Institutional EMR	Single (organ-space SSI)	Single algorithm	Regularized Bayesian multilevel logistic regression
Betts et al [89] (2019)	Australia	2009 to 2015	Multiple Medical Centers	Obstetric	Perinatal data collection and Queens Hospital admitted patient data collection	Single (composite SSI)	Single algorithm	XGBoost
Bülow et al [127] (2022)	Sweden	2008 to 2015	Multiple Medical Centers	Orthopedics	The Swedish hip arthroplasty register data	Single (periprosthetic joint infection [PJI])	Single algorithm	LASSO Regression
Cui et al [128] (2025)	China	2013 to 2024	Multiple Medical Centers	Orthopedics	Institutional EHR	Single (composite SSI)	Five algorithms	GBM^s^
Hopkins et al [90] (2020)	United States	2000 to 2015	A single medical center	Orthopedics	Institutional EHR	Single (composite SSI)	Single algorithm	DNN
Li and Yan [129] (2024)	China	2018 to 2021	A single medical center	Gastrointestinal	Institutional EHR	Single (composite SSI)	Three algorithms	DL^t^
Liu et al [130] 2022	China	2010 to 2019	A single medical center	Orthopedics	Institutional EHR	Not reported	Six algorithms	XGBoost
Yeo et al [131] (2023)	United States	2016 to 2019	A single medical center	Orthopedics	Institutional EHR	Single (composite SSI)	Five algorithms	ANN
Hui et al [132] (2023)	China	2018 to 2022	A single medical center	Orthopedics	Institutional EHR	Single (composite SSI)	Ten algorithms	ET^u^
Liang [134] et al (2025)	China	2021 to 2022	A single medical center	Gastrointestinal	Institutional EHR	Single (superficial SSI)	Eight algorithms	RF
Liao et al [91] (2018)	China	2017	A single medical center	Neurosurgery	Institutional EHR	Not reported	Single algorithm	ANN
Hu et al [108] (2024)	China	2018 to 2023	A single medical center	Gastrointestinal	Institutional EHR	Not reported	Two algorithms	DT^v^
Cheng et al [135] (2024)	China	2018 to 2020	A single medical center	Thoracic	Institutional EHR	Single (composite SSI)	Six algorithms	Meta - LASSO Regression
Gutierrez-Naranjo et al [106] (2024)	United States	2014 to 2020	A single medical center	Orthopedics	Institutional EHR	Single (composite SSI)	Four algorithms	Not specified
Wang et al [96] (2025)	China	2020 to 2022	A single medical center	Gastrointestinal	Institutional EHR	Single (composite SSI)	Single algorithm	LASSO Regression
Song and Wei [133] (2025)	China	2023 to 2024	A single medical center	Orthopedics	Institutional EHR	Not reported	Single algorithm	RF
An et al [136] (2023)	China	2017 to 2021	A single medical center	Orthopedics	Institutional EHR	Single (composite SSI)	Single algorithm	LASSO Regression
Choi et al [137] (2024)	Korea	2010 to 2021	Multiple Medical Centers	Orthopedic	Health insurance review and assessment service data	Not reported	Single algorithm	RF
Pang et al [138] (2025)	China	2017 to 2025	Multiple Medical Centers	Orthopedics	Institutional EHR	Not reported	Ten algorithms	SVM
Ghabisha et al [139] (2026)	Yemen	2018 to 2023	A single medical center	Gastrointestinal	Institutional EHR	Not reported	Four algorithms	XGBoost
Cao et al^w^ [77] (2026)	China	2020 to 2024	Multiple medical centers	Gastrointestinal	Institutional EHR	Single (organ-space SSI)	Seven algorithms	Stacking ensemble models
De et al [93] (2025)	India	Not reported	Not reported	Multiple surgical procedures	Not reported	Not reported	Four algorithms	RF
Michael et al [115] (2025)	United States	2017 to 2021	Multiple medical centers	Gastrointestinal	Trauma Quality Improvement Program (TQIP) dataset	Single (composite SSI^x^)	Five algorithms	XGBoost
Haider et al [116] (2025)	Japan	2010 to 2024	Multiple medical centers	Orthopedics	Institutional EHR	Single (composite SSI)	Seven algorithms	Stacking ensemble model
Orlandi et al [140] (2022)	Brazil	2017 to 2019	Multiple medical centers	Cardiac	REPLICCAR II database	Single (organ-space SSI)	Two algorithms	LASSO Regression
Kocbek et al [94] (2019)	Norway	2004 to 2012	A single medical center	Gastrointestinal	Institutional EHR	Not reported	Three algorithms	LASSO Regression
Gowd et al [107] (2019)	United States	2005 to 2017	Multiple medical centers	Orthopedics	ACS-NSQIP data	Not reported	Six algorithms	Logistic Regression
Nie et al [141] (2026)	China	2020 to 2024	Two medical centers	Orthopedics	Institutional EMR	Not reported	Eight algorithms	RF
Jha et al [105] (2026)	India	Not reported	A single medical center	Gastrointestinal	Institutional EMR	Not reported	Five algorithms	SVM
Rahimi et al [142](2025)	Iran	2017 to 2024	A single medical center	Gastrointestinal	Institutional EHR	Single (Composite SSI)	Four algorithms	RF
Ying et al [143] (2026)	China	2011 to 2014	A single medical center	Orthopedics	Institutional EHR	Not reported	Six algorithms	LightGBM
Zhang et al [144] (2025)	China	2023 to 2024	A single medical center	Orthopedics	Institutional EHR	Not reported	Eight algorithms	GBM
Zhou et al [145] (2025)	China	2015 to 2020	A single medical center	Head and neck surgery	Institutional EHR	Not reported	Two algorithms	RF
Li et al [92] (2025)	China	2022 to 2023	A single medical center	Orthopedics	Institutional EHR	Single (deep SSI)	Single algorithm	DT
Diagnostic Prediction (n=19)								
Verberk et al [85] (2023)	Sweden	2015 to 2020	A single medical center	Gastrointestinal	Institutional EHR	Single (composite SSI)	Two algorithms	DL
Petrosyan et al [29] (2021)	Canada	2010 to 2015	A single medical center	Multiple surgical procedures	The discharged abstract database and same day surgery database; The physician services database; The Ontario health insurance plan database	Single (composite SSI)	One algorithm plus three predictor sets per outcome	RF
Zhu et al [100] (2021)	United States	2011 to 2017	Two medical centers	Multiple surgical procedures	ACS-NSQIP data	Multiple SSIs (including composite SSI, superficial SSI, and organ-space SSI)	Single algorithm	LASSO Regression
Colborn et al [99] (2023)	United States	2013 to 2019	Multiple medical centers	Multiple surgical procedures	Institutional EHR	Multiple postoperative infections (including composite SSI^x^)	Single algorithm	Regularized Logistic Regression
Kiser et al [84] (2024)	United States	2016 to 2021	A single medical center	Multiple surgical procedures	Enterprise data warehouse (only using EHR)	Single (composite SSI)	Six algorithms	LSTM^y^
Colborn et al [99] (2018)	United States	2013 to 2016	A single medical center	Multiple surgical procedures	Health data compass (a data warehouse composed of EHR)	Postoperative infection (including composite SSI)	Nine algorithms	LASSO Regression
Ruan et al [146] (2022)	United States	2006 to 2018	A single medical center	Gastrointestinal	ACS-NSQIP data	Multiple SSIs (including superficial, wound infection, and organ-space SSI)	Four algorithms plus three predictor sets per outcome	Multiple GRU-D Model
Weller et al [83] (2018)	United States	2010 to 2013	A single medical center	Gastrointestinal	Institutional EHR	Multiple postoperative complications (The type of SSI is not reported)	Five algorithms	LASSO Regression
Rennert-May et al [103] (2022)	Canada	2013 to 2019	Multiple medical centers	Cardiac	Alberta Health Services System	Single (composite SSI)	Two algorithms	Regularized Logistic Regression
Flores-Balado et al [39] (2023)	Spain	2014 to 2021	Multiple medical centers	Orthopedics	Institutional EHR; Clinical notes	Single (composite SSI)	Single algorithm	XGBoost
Kalisnik et al [147] (2025)	Germany	2007 to 2022	A single medical center	Cardiac	Quality management SAP; THG-QIMS database	Single (deep sternal wound infection [DSWI])	Two algorithms	XGBoost
Yu et al [104] (2014)	China	2005 to 2008	Two medical centers	Cardiac	National health insurance claims data; Health care-associated infection Surveillance data	Not reported	Three algorithms	DT
Xu et al [148] (2020)	China	2015 to 2016	Multiple medical centers	Gastrointestinal	Duke Infection Control Outpatient Surveillance Network (DICOSN)	Not reported	Five algorithms	RF
Cao et al^w^ [77] (2026)	China	2020 to 2024	Multiple medical centers	Gastrointestinal	Institutional EHR	Single (organ-space SSI)	Seven algorithms	Stacking ensemble model
Li et al [102] (2025)	China	2011 to 2024	A single medical center	Orthopedics	Institutional EMR	Single (composite SSI)	Single algorithm	LASSO regression
Celik et al [87] (2025)	United States	2018 to 2023	A single medical center	Gastrointestinal	Institutional EHR	Not reported	Three algorithms	XGBoost
Perkins et al [149] (2024)	United States	2020 to 2022	A single medical center	Multiple surgical procedures	Institutional EHR	Single (composite SSI)	Single algorithm	LASSO regression
Phuyal et al [150] (2026)	United States	2016 to 2022	Multiple medical centers	Breast surgery	ACS-NSQIP Data	Single (composite SSI)	Single algorithm	XGBoost
Agostinho et al [86] (2025)	Sweden	2016 to 2022	A single medical center	Multiple surgical procedures	Institutional EHR	Single (composite SSI)	Six algorithms	NB and DNN

a SSI: surgical site infection.

b ACS-NSQIP: American College of Surgeons National Surgical Quality Improvement Program.

c Composite SSI including superficial, deep, and organ-space SSIs.

d DNN: deep neural network.

e SVM: support vector machine.

f ANN: artificial neural network.

g EMR: electronic medical record.

h EHR: electronic health record.

i LASSO: least absolute shrinkage and selection operator.

j Composite SSI including superficial and deep SSIs

k NB: native Bayes.

l Composite SSI including deep and organ SSIs

m BPMI: Bayesian-Probit regression model with multiple-imputation.

n RF: random forest.

o NN: neural network.

p HBGT: histogram-based gradient boosting.

q XGBoost: extreme gradient boosting.

r STS ACSD: The Society of Thoracic Surgeons Adult Cardiac Surgery Database.

s GMB: gradient boosting machine.

t DL: deep learning.

u ET: extra trees classifier.

v DT: decision tree.

w the two records are from the same study, which developed both prognostic and diagnostic prediction

x Composite SSI including superficial, deep, organ-space SSIs, and wound disruption.

y LSTM: long short-term memory.

### Data-Level Explainability

All included models used structured data, typically extracted from electronic health records or registry databases. These predictors were organized into 21 clinically meaningful categories spanning patient, surgery, laboratory, and clinical intervention factors across preoperative, intraoperative, and postoperative phases. Interrater agreement for this classification was high (Cohen κ=0.868). The operational definitions for each category are provided in Multimedia Appendix 2. Only 8 models included free-text data from intraoperative and postoperative clinical records [39,81-87].

Figure 2 illustrates the distribution of variable categories and corresponding collection periods. Among prognostic models, the most frequently represented feature categories were health status and lifestyle collected preoperatively (60/72, 83.3%), followed by individual demographics (57/72, 79.2%), and surgical process details recorded during the surgeries (57/72, 79.2%). Surgery setting information and technique were also regularly used (49/72, 68.1%; 39/72, 54.2%). Other commonly used preoperative features included comorbidity situation (50/72, 69.4%) and inflammatory laboratory indicators (33/72, 45.8%). Features collected postoperatively were generally less frequently represented, which were only included in 11 models across 9 studies [29,88-92,152], and mainly involved recovery status after surgery (9/72, 12.5%) and admission status (5/72, 6.9%). Two studies [93,94] developed predictive models using laboratory test results alone. One incorporated laboratory test results obtained at three distinct time points during the perioperative period [93], while the other included only preoperative laboratory results [94]. Nevertheless, 6 models constructed in 2 studies [95,96] did not specify the timing of laboratory testing and vital-sign measurement.

Among the diagnostic models, the frequently represented categories included surgical process details (13/26, 50%), administrative codes obtained after surgery (10/26, 38.5%), individual demographics (10/26, 38.5%), and postoperative clinical diagnostic orders and treatments (9/26, 34.6%). Across the eight laboratory test categories, the proportion of diagnostic models incorporating intraoperative or postoperative laboratory results ranged from 11.5% to 23.1%, whereas none of the prognostic models incorporated such data. Five studies relied exclusively on postoperative predictors, most commonly laboratory tests and clinical interventions [29,99-102]. Two studies developed models based on *ICD-9* (*International Classification of Diseases, Ninth Revision*) or *ICD-10* (*International Statistical Classification of Diseases, Tenth Revision*) administrative codes alone [84,103]. Additionally, one study developed diagnostic models using longitudinal laboratory data collected from 4 days before surgery to 14 days after surgery [99]. The timing of predictor collection could not be determined for three models because of insufficient reporting [39,87,104].

**Figure 2.**
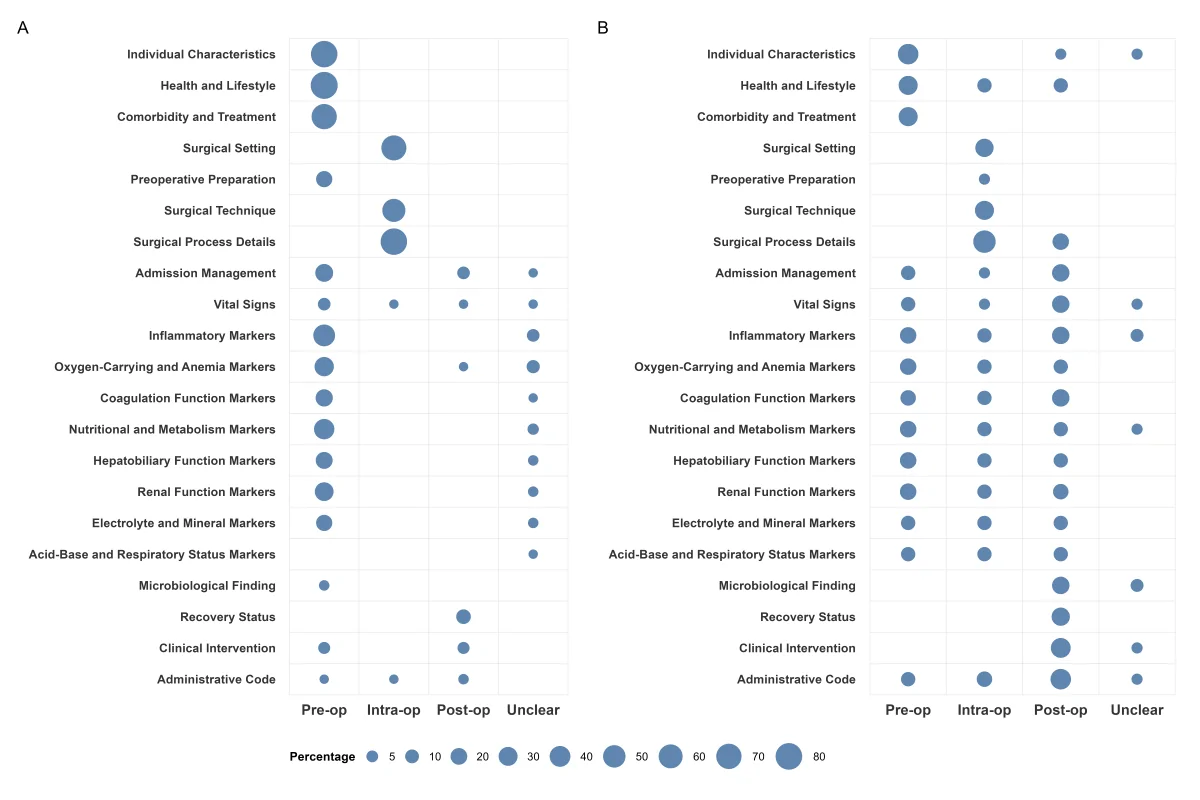
Bubble plot of predictor categories and collection period in prognostic and diagnostic models. Predictor categories are displayed on the y-axis and data collection periods on the x-axis for (A) prognostic models and (B) diagnostic models. Bubble size represents the percentage of models within the corresponding model type that incorporated each category-period combination, as indicated in the legend. Because individual models could include predictors from multiple categories and collection periods, the percentages are not mutually exclusive. “Unclear” indicates that the collection period was not reported or could not be determined. Pre-op: preoperative period; Intra-op: intraoperative period; Post-op: postoperative period.

### Methodological Explainability

Figure 3 shows the frequency of explainability techniques used across different ML model categories. Overall, 63.3% (62/98) of predictive models were black-box models, with ensemble learning models ranking first (38/62, 61.3%), followed by deep learning (20/62, 32.3%). Ensemble learning models include tree-based bagging, boosting, and heterogeneous stacking. Only 4 black-box models were based on kernel structure. Among these black-box models, 15 models did not specify the explicit explanatory approaches used to produce their explanatory outcomes. Among the 47 models that reported explanatory approaches, the Shapley additive explanation (SHAP) was the most frequently applied method (n=27), followed by PFI (n=7). Specifically, the combination of SHAP values and ensemble learning models was the most common combination among black-box models reporting specific explanatory approaches (18/47, 38.3%), followed by SHAP values combined with deep learning models (8/47, 17%) and PFI used in ensemble learning models (4/47, 8.5%).

In addition to post hoc explanation methods, two ensemble learning studies used the built-in interpretability of extreme gradient boosting (XGBoost): one assessed feature importance based on how often each feature was used for splitting across trees [97], whereas the other did not specify an importance metric [98]. Three studies explained their models using feature coefficients estimated by regression models [82,90,105]. Two studies used frequency-based measures: one kernel model counted the occurrences of free-text terms in the model [81], while the other ensemble learning model recorded the frequency of each feature identified as important across repeated random forest models [79]. One deep learning study incorporated an attention layer to enhance the model’s intrinsic explainability [84].

Among the remaining 36 inherently interpretable models, 61.1% (22/36) were regression-based models, followed by Bayesian models (8/36, 22.2%) and decision tree models (6/39, 16.7%). Six models did not report any explanatory approaches or explainability metrics. Among the regression-based models, 18 provided the coefficient of each feature as an explanation, while one model was explained based on the frequency with which each variable was included across 100 cross-validation runs [106]. Three regression models did not report the specific explanation methods or importance metric used [106,107]. Four decision tree models used Gini impurity values or feature splitting location to explain feature importance [92,102,104,108], and one calculated the frequency of free text words [85,108]. Additionally, 3 Bayesian models were explained using SHAP plots, which allowed their feature importance to be compared with other ML models developed in the same paper [86,109,110]. Two Bayesian models were combined with a regression model and hence used coefficients to illustrate the feature importance [111,112].

**Figure 3.**
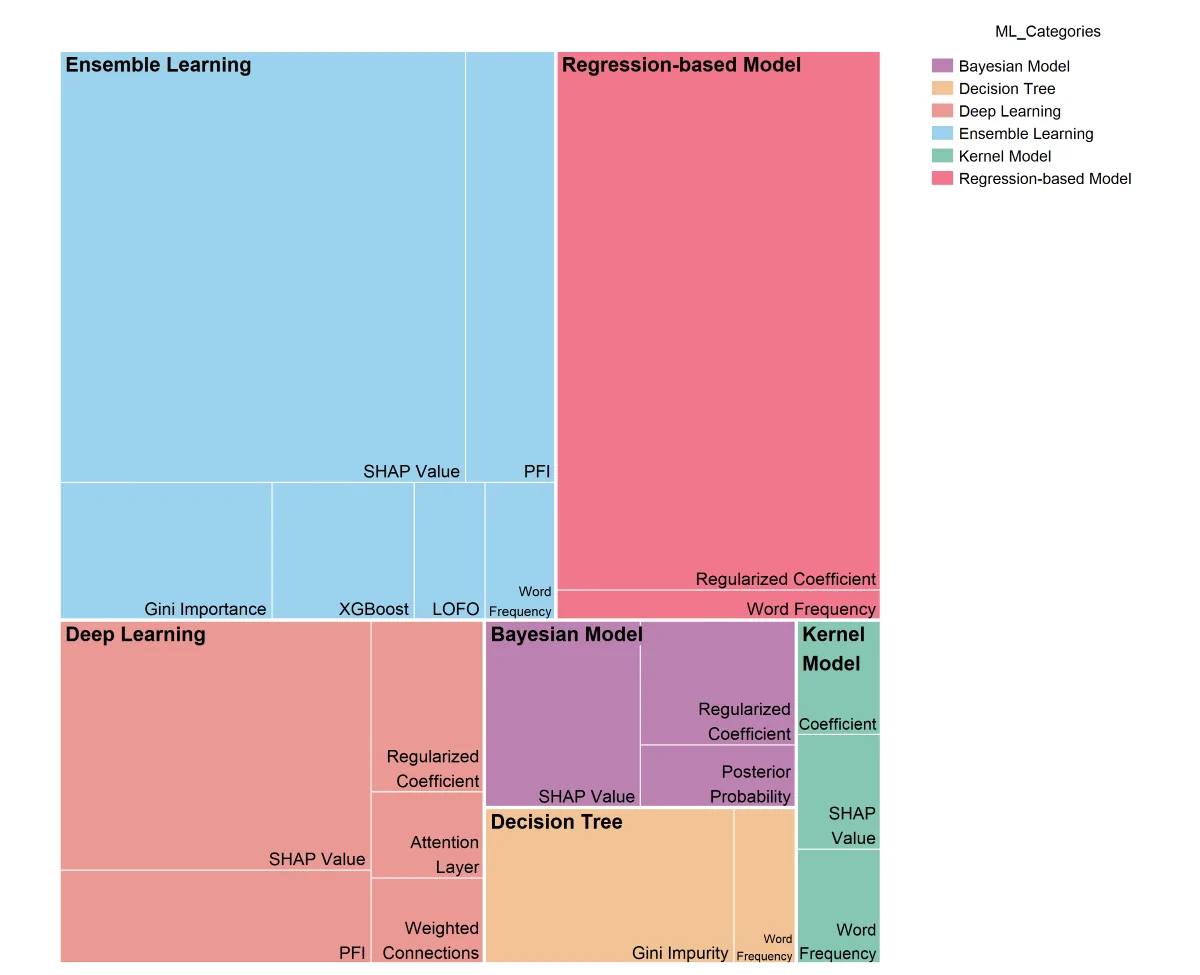
Distribution of explainable techniques used in different machine learning algorithms. The tree map shows the corresponding relationships between explainable techniques and ML models. Segment size indicates the frequency of each relationship, and the colors differentiate machine learning categories, as indicated by the legend. LOFO: leave-one-feature-out; PFI: permutation feature importance; DT: decision tree; ML: machine learning; SHAP: Shapley additive explanations; XGBoost: extreme gradient boosting.

### Output-Level Explainability

Explanations were usually presented as static visualizations (95/101, 94.1%) to show the rank of feature importance or model weight, such as bar charts, heatmaps, word clouds, and tables. Six models established interactive tools, such as nomograms or risk calculators [78,111,113,152].

Figure 4 shows how frequently each predictor category was identified as important in models using multiple variable categories, stratified by SSI type. Eight models using variables from a single category are described separately. In prognostic models, 317 important features were identified for composite SSI, 38 each for superficial and deep SSI, and 55 for organ-space SSI. The most frequently reported important features were related to health status and lifestyle across all four SSI types, with 14.8% (47/317) in composite SSI, 23.7% (9/38) in superficial SSI, 31.6% (12/38) in deep SSI, and 20% (11/55) in organ-space SSI. Beyond this common pattern, the distribution of other important predictor categories varied across SSI types. For composite SSI, surgical process details accounted for 13.8% (44/317) of important features, followed by surgical technique (42/317, 13.2%) and comorbidity and treatment intervention (33/317, 10.4%). For superficial SSI, the second most common top predictors were individual characteristics and comorbidities and treatment (both 6/38, 15.8%), followed by surgical process details and nutritional and metabolism markers (both 4/38, 10.5%). The critical features for deep SSI prediction consistently focused on comorbidity and treatment (7/38, 18.4%), individual characteristics (5/38, 13.2%), and surgical setting (3/38, 7.9%). For organ-space SSI, the second most common key predictor category was surgical setting (10/55, 18.2%), followed by comorbidities and treatment (7/55, 12.7%). Among those models for unclassified SSI type, inflammatory markers were the most common key predictor classification (20/107, 17.7%), followed by surgical process details (17/107, 15.9%) and nutritional and metabolism markers (13/107, 11.5%). Zero-importance predictor categories varied across 5 SSI types and predominantly involved laboratory indicators.

In diagnostic models, 83 features were identified as important for composite SSI, 12 for superficial SSI, 10 for deep SSI, and 27 for organ-space SSI. Postoperative features were more prominent. For composite SSI, clinical interventions were the most frequently reported category, comprising 26.5% (22/83) of important features, followed by administrative codes (18/83, 21.7%) and recovery status (10/83, 12%). For superficial SSI, vital signs, clinical interventions, and administrative codes were most common (2/12, 16.7% each). For deep SSI, recovery status ranked first (3/10, 30%), followed by surgical process details (2/10, 20%). For organ-space SSI, vital signs (8/27, 29.6%) were widely identified as important predictors, followed by surgical process details and clinical interventions (3/27, 11.1% each). For SSI without clear classification, preoperative and intraoperative metrics, especially health and lifestyle (8/36, 22.2%) and surgical process details (7/36, 19.4%), were the most common predictors. The distribution of zero-importance feature categories varied across SSI types, with patient demographics, health and lifestyle, comorbidities, and laboratory indicators most frequently assigned zero importance.

Two studies [29,103] developed diagnostic models using administrative codes alone. A total of 53 important codes were used and could be grouped into 10 clinically meaningful categories, involving postoperative complications, iatrogenic injuries, laboratory biomarkers, inpatient services, therapeutic or nutritional interventions, surgical procedures, gastrointestinal diseases, malignant neoplasms, and other systemic diseases. Postoperative complications were the most frequent category, followed by surgical procedures and abdominal diseases. Additionally, one study comprehensively assessed the importance of the preoperative collection period and variables by examining selection frequencies and found that the leukocyte count measured during the medium interval was most important [93]. Another study compared the importance of inflammatory markers measured at 3 collection times during the perioperative period and found that those measured 3 days after surgery were more critical [94].

**Figure 4.**
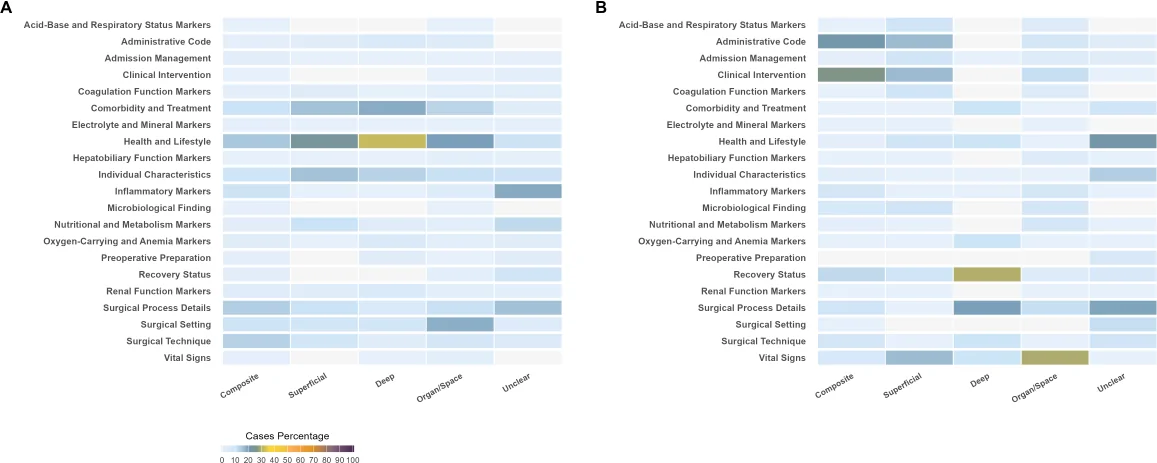
Frequency distribution of critical features across surgical site infections categories in prognostic model and diagnostic model. Heat maps show the distribution of predictive features in the included studies, stratified by surgical site infections categories, across prognostic model and diagnostic model. The left one (A) indicates the figure of prognostic model, while the right one (B) indicates the diagnostic model. The color gradient denotes the percentage of cases, increasing from light blue (0%) to dark purple (100%), while light grey represents corresponding categories that are not initial input features.

### Risk of Bias and Applicability

The interrater agreement between the 2 researchers (RS and YL) was high, with the overall Cohen κ of 0.863 (*P*<.01), and domain-level values ranging from 0.319 to 1. Detailed results are shown in Figure 5 and Multimedia Appendix 3.

The 77 included studies [29,30,39,77-150] comprised 98 eligible models for PROBAST+AI assessment. As one study reported model evaluation alone [114], 98 model development processes and 97 model evaluation processes were ultimately assessed [153].

For model development, 92.8% (91/98) of models were judged to have an overall high-quality concern, and only 7 models had an overall unclear rating [78,80,86,115,116]. Overall, 88.8% of models (87/98) were assessed as high-quality concerns in domain 1 (participants), which was the primary driver of great concern, followed by domain 4 (analysis, 76.5%, 75/98). In domain 1, concerns were mainly related to inappropriate or unclear study design. In domain 4, concerns are often derived from small development datasets relative to model complexity and the absence of a procedure for handling missing data. Most of domain 2 (predictors) and domain 3 (outcomes) were rated as unclear, with 58.2% (57/98) and 44.9% (44/98), respectively. The main issues were unclear definitions of predictors and outcomes, uncertain prediction horizons, and incomplete reporting of measurement and assessment procedures.

For model evaluation, 97.9% (95/97) of models were classified as high risk of bias, and 2 models were evaluated as having an unclear risk [115]. In development, domains 1 (high risk: 86/97, 88.7%) and 4 (high risk: 86/97, 88.7%) were the main contributors to elevated risk. Most models were internally evaluated, carrying over limitations in design and data preprocessing. Additionally, model performance assessment was largely limited to discrimination, with calibration and clinical utility rarely evaluated, resulting in an incomplete evaluation of model performance. The rating distributions and underlying reasons for domains 2 and 3 during model evaluation were identical to those during model development.

Approximately 62.2% (61/98) of models had low applicability concerns in model applicability, whereas 18 models presented unclear concerns due to ambiguous predictor measurement and outcome assessment procedures.

**Figure 5.**
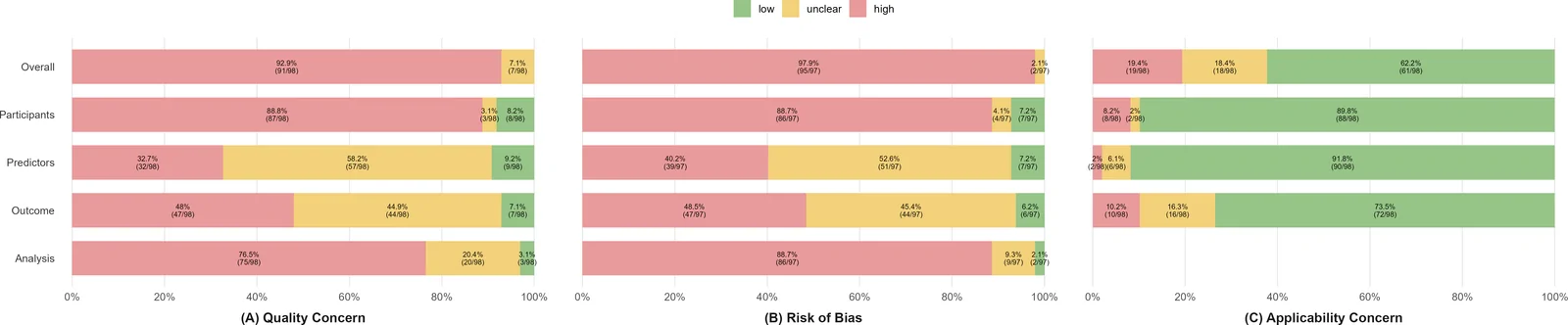
Summary of risk of bias, quality concern, and applicability concern assessed with PROBAST+AI (Prediction model Risk Of Bias Assessment Tool for prediction models using regression or artificial intelligence). Percentage stacked bar charts summarize ratings of model risk of bias, quality concern, and applicability concern. Numbers on the x-axis (0-100) indicate the proportion (%) of models in each category. Green denotes low risk or concern; yellow denotes risk or concern; red denotes high risk or concern.

## Discussion

### Principal Findings

This scoping review mapped explainability in ML models for SSI prediction across the data, methodological, and output levels. Previous reviews have shown that explainable ML has the potential to improve SSI surveillance and support earlier clinical action by integrating large and complex health data [35,36]. However, the clinical implementation of ML models depends not only on predictive performance but also on whether clinicians can understand, trust, and act on the explanations they provide.

Overall, explainability practices in ML-based SSI prediction remain in their early stages, with important information gaps across all three levels. At the data level, existing models incorporated predictors from multiple perioperative domains. However, unclear definitions and collection timing limited the assessment of their clinical meaning and suitability for the intended prediction point. At the method level, black-box models commonly use post-hoc techniques such as SHAP and PFI. Inherently interpretable models generally relied on their internal structures, while some studies supplemented them with post-hoc techniques to form hybrid explanation strategies. Nevertheless, some studies did not clearly report the explanation methods used. Available reports also provided limited information on method-selection rationales and intended explanatory purposes, making it difficult to assess compatibility with model architectures and application contexts. At the output level, explanations were dominated by static visualizations of global feature importance, with limited evidence regarding causality, actionability, or user-level effectiveness. Many top-ranked predictors represented inherent patient characteristics or clinical conditions rather than clearly modifiable factors, further limiting their direct translation into clinical interventions. The generally high risk of bias further restricted the credibility and generalizability of these explanations. Thus, current ML explanations help describe model behavior, but available evidence remains insufficient to establish their clinical context and practical utility.

### Predictor Selection and Clinical Relevance

The pattern of predictor selection differed meaningfully between prognostic and diagnostic models, with important clinical implications. Prognostic models were dominated by preoperative and intraoperative variables, especially patient characteristics, lifestyle factors, and surgical process details. This is appropriate for models intended to estimate risk when preventive strategies are still being implemented. Diagnostic models, by contrast, more often rely on postoperative variables, particularly recovery status and clinical interventions, which is consistent with their purpose of identifying SSI after it has developed.

Even so, the current evidence suggests that prognostic models may be missing clinically informative postoperative signals. SSI commonly occurs 10 to 20 days after surgery, and many cases emerge after discharge [154,155]. Early postoperative trends in vital signs [156], inflammatory markers [153,157], and recovery status [158] may therefore improve the timeliness and precision of prediction. This suggests that future prognostic models should move beyond static perioperative snapshots and incorporate dynamic postoperative trajectories when clinically appropriate. In parallel, important contextual factors such as operating room environment remain underrepresented, despite evidence that they may contribute to SSI risk [159,160].

### Ambiguity in Predictor Definitions and Timing

Several studies did not clearly report the definitions and collection timing of model predictors, which may pose a major barrier to clinical interpretability and actionability. This lack of chronological clarity regarding multidomain features introduces serious uncertainty about whether these predictors would be available at the intended point of clinical use and whether they are clinically plausible drivers of SSI risk, ultimately leading to an unclear definition of model deployment windows and limiting their real-world clinical application [161,162]. This methodological problem is particularly important for potential longitudinal variables such as laboratory indicators and vital signs, whose clinical meaning and normal ranges can vary substantially depending on the exact time of measurement [157,159,163].

Administrative codes were relatively common in diagnostic models since they are readily available in retrospective datasets. However, they primarily reflect billing practices, institutional workflows, and reimbursement incentives rather than straightforward clinical observations [164,165]. Our findings revealed that most models incorporating administrative codes rarely report their context, including the exact temporal alignment of each medical order. Without explicit temporal records, the codes may include medical interventions targeted at SSI diagnosis and treatment, thereby introducing information leakage or circular reasoning [166] and weakening both reproducibility and cross-institutional interpretability. These findings underscore the need for more standardized reporting of predictor semantics and collection timing.

### Selection and Alignment of Explainability Methods

The review found that combinations of ML models and explanatory approaches are considerably diverse, with no consistent correspondence. Among inherently interpretable models, most explanations matched the models’ own structures, which are more transparent because they directly reflect their internal logic [47]. A few models adopted hybrid explanation strategies by applying post hoc tools to inherently interpretable models. This strategy facilitates cross-model comparison within a study; however, post hoc explanations may not fully capture the internal logic of inherently interpretable models, thereby obscuring some of their intrinsic transparency [47,167].

For black-box models, aside from a few that attempted to reform the model structure with interpretable modules, most used post hoc explanatory approaches. SHAP and PFI were the most common methods. However, it is noteworthy that these methods answer different questions, and that distinction is often not made explicit. SHAP estimates how a feature contributes to a specific prediction [168], whereas PFI measures how model performance changes when a feature is shuffled [169]. Clinically, SHAP is often more meaningful because it links predictors to individual risk estimates, whereas PFI mainly reflects the model’s dependence on a feature. In our review, many studies reported “feature importance” without clarifying the questions the explanation method was intended to answer, which can mislead clinicians and limit practical use. Future studies should therefore consider explainability techniques based on the clinical purpose of the explanation.

Furthermore, the model’s intrinsic structure influences the fidelity of post hoc explanatory methods. This issue is especially important for SHAP. Although SHAP is widely used in SSI prediction, its reliability depends on the underlying model. For tree-based models, TreeSHAP can closely reflect the model’s decision structure [170]. However, in deep learning, feature interactions are often nonlinear and distributed, making additive explanations less faithful and more sensitive to assumptions such as the choice of baseline [171,172]. Because these differences are rarely discussed in SSI studies, SHAP-derived outputs are often presented as if they were interchangeable across architectures. Greater attention to method-model alignment is therefore needed to improve the fidelity of explanations.

Current explainable methods primarily provide feature importance rankings or graphical visualizations. These outputs indicate which predictors contributed more but often provide limited context on the meaning and actionability of the explanations [173]. Emerging studies have shown that transforming model outputs into human-centered narrative explanations can improve clinicians’ trust and acceptance of AI-assisted decision support [174-176]. Future research should therefore move beyond technical explainability and focus on human-centered explainability. Additionally, incorporating a large language model (LLM) into explanatory approaches, which excels at generating natural and conversational language [177], could assist in translating narratively technical outputs into natural language. Additionally, co-development with clinicians and model developers to achieve participatory system design [176,178] may help bridge the gap between explainability and clinical usability [179].

### Clinical Interpretation of Feature Importance

A clinically important finding was the consensus on critical predictors across SSI subtypes, which largely aligns with established SSI risk factors [180-183]. These recurrent predictors may help minimize optimal feature sets and guide future model development [169], thereby providing some reassurance about the face validity of the models. However, since most prediction models were developed using observational data, these feature-importance outputs should be interpreted as associations rather than causal effects. Without causal analysis, there is a risk that nonmodifiable correlates will be mistaken for actionable targets, which may lead to inappropriate clinical inference [184,185]. Beyond ranking important features, future studies should therefore clinically validate model-identified features by assessing their clinical plausibility, causal relevance, modifiability, and actionability in clinical decision-making [179,186]. Counterfactual inference approaches may be better suited for this purpose [187-189].

The apparent lack of importance of several laboratory indicators is somewhat counterintuitive given their clinical relevance after SSI onset [153,157]. However, this may reflect either limited independent predictive value or methodological limitations in the explanation process. Common problems such as class imbalance, collinearity, and incomplete feature engineering can distort feature rankings and obscure relevant predictors [190-192]. Additionally, it is shown that the values and meanings of biomarkers can vary substantially across the perioperative course [193-195], and some eligible studies in this review have shown that the importance of laboratory tests collected from different time windows varied significantly [93,94]. However, the timing of the laboratory test was reported only as an implicit period rather than an exact time in most included studies, as previously discussed. These features may therefore appear insignificant simply because they were measured at an inappropriate time or evaluated within an unsuitable modeling framework. Future studies should report collection timing clearly, assess temporal sensitivity, and address collinearity explicitly to ensure that explanatory outputs are clinically credible [41].

### Limitations

This review has several limitations. First, one protocol amendment should be acknowledged: the omission of one planned subgroup analysis because some SSI categories contained too few models for meaningful comparison. Second, when multiple models were reported in a study, we extracted explainability information only for the model the authors selected as best performing. Because predictive accuracy and explainability are not always aligned, this may have excluded informative alternative models. Third, the definition of “best-performing” varied across studies, which limited direct comparisons. Fourth, this is a rapidly evolving field, so the review may not capture the most recent developments. Finally, because no established framework exists for quantitatively synthesizing feature-importance outputs across heterogeneous explainability methods, we summarized our findings qualitatively rather than meta-analyzing them.

### Conclusions

To our knowledge, this review is the first to focus on how model explainability is implemented and reported in this field. We systematically mapped current practices across three complementary clinical levels: predictors, explanatory techniques, and outputs. The findings revealed incomplete explainability information across all three levels, with current practices remaining largely limited to static, global feature attribution. This review provides baseline evidence on implementation and reporting gaps in explainable ML for SSI prediction and identifies priority dimensions for future evaluation frameworks. Further studies should strengthen reporting at each level and involve clinicians and other intended users in developing and validating explainability evaluation approaches tailored to real-world settings.

## Supplementary material

10.2196/94617 Multimedia Appendix 1 Search strategy.

10.2196/94617 Multimedia Appendix 2 Feature categories.

10.2196/94617 Multimedia Appendix 3 Risk of bias assessment result.

10.2196/94617 Checklist 1 PRISMA-ScR fillable checklist.

10.2196/94617 Checklist 2 PRISMA_2020_abstract_checklist.

## Abbreviations

ACS-NSQIP: American College of Surgeons National Surgical Quality Improvement Program
AUC: area under the receiver operating characteristic curve
CDC: Centers for Disease Control and Prevention
CHARMS: Checklist for Critical Appraisal and Data Extraction for Systematic Reviews of Prediction Modelling Studies
DSWI: deep sternal wound infection
*ICD-10*: *International Statistical Classification of Diseases, Tenth Revision*
*ICD-9*: *International Classification of Diseases, Ninth Revision*
JBI: Joanna Briggs Institute
LLM: large language model
LSTM: long short-term memory
ML: machine learning
NB: native Bayes
NHSN: National Healthcare Safety Network
PFI: permutation feature importance
PRISMA: Preferred Reporting Items for Systematic Reviews and Meta-Analyses
PRISMA-S: Preferred Reporting Items for Systematic Reviews and Meta-Analyses literature search extension
PRISMA-ScR: Preferred Reporting Items for Systematic Reviews and Meta-Analyses extension for Scoping Reviews
PROBAST+AI: Prediction Model Risk of Bias Assessment Tool for Prediction Models Using Regression or Artificial Intelligence
ROB: risk of bias
SHAP: Shapley Additive Explanations
SSI: surgical site infection
WHO: World Health Organization
XGBoost: extreme gradient boosting
