# Supplementary material for: Explainable Machine Learning Predictive Models for Surgical Site Infections: Scoping Review
Source: J Med Internet Res. 2026 Sep 30;28:e94617. doi: 10.2196/94617 (PMC13626550; doi:10.2196/94617)
Supplement: Supplementary file 1 [file jmir-v28-e94617-s001.docx]

**Multimedia Appendix 2.** Search strategy.

1. **Results of update search**

**PubMed**

| PubMed (date searched: January 1, 2010 to July 14, 2026): | | |
| --- | --- | --- |
|  |  |  |
| 1 | "Artificial Intelligence"[MeSH] OR "Machine Learning"[MeSH] OR "Deep Learning"[MeSH] OR "Supervised Machine Learning"[MeSH] OR "Unsupervised Machine Learning"[MeSH] OR "Neural Networks, Computer"[MeSH] OR "Support Vector Machine"[MeSH] OR "Random Forest"[MeSH] OR "Decision Trees"[MeSH] OR "Boosting Machine Learning Algorithms"[MeSH] OR "Bayes Theorem"[MeSH] OR "Data Mining"[MeSH] | 316307 |
| 2 | "artificial intelligence"[tiab] OR "machine learning"[tiab] OR "deep learning"[tiab] OR "data mining"[tiab] OR "neural network*"[tiab] OR "artificial neural network*"[tiab] OR ANN[tiab] OR "convolutional neural network*"[tiab] OR CNN[tiab] OR "recurrent neural network*"[tiab] OR RNN[tiab] OR transformer*[tiab] OR "support vector*"[tiab] OR SVM[tiab] OR "random forest*"[tiab] OR "decision tree*"[tiab] OR "gradient boost*"[tiab] OR XGBoost[tiab] OR AdaBoost[tiab] OR LightGBM[tiab] OR CatBoost[tiab] OR "naive bayes"[tiab] OR bayesian[tiab] OR "k-nearest neighbor*"[tiab] OR KNN[tiab] OR "ensemble learning"[tiab] OR "ensemble method*"[tiab] OR "supervised learning"[tiab] OR "unsupervised learning"[tiab] OR "reinforcement learning"[tiab] OR "transfer learning"[tiab] OR "LASSO"[tiab] OR (classifi*[tiab] AND algorithm*[tiab]) | 635319 |
| 3 | #1 OR #2 | 708893 |
| 4 | "Surgical Wound Infection"[MeSH] OR "Wound Infection"[MeSH] | 24760 |
| 5 | "surgical site infection*"[tiab] OR SSI[tiab] OR SSIs[tiab] OR "surgical wound infection*"[tiab] OR "postoperative wound infection*"[tiab] OR "postoperative infection*"[tiab] OR "post-operative infection*"[tiab] OR "post operative infection*"[tiab] OR "postsurgical infection*"[tiab] OR "post-surgical infection*"[tiab] OR "incisional infection*"[tiab] OR "incision site infection*"[tiab] OR "operative wound infection*"[tiab] OR "wound infection*"[tiab] | 49260 |
| 6 | #4 OR#5 | 58741 |
| 7 | "Risk Assessment"[MeSH] OR "Models, Statistical"[MeSH] OR "Decision Support Techniques"[MeSH] | 557580 |
| 8 | predict*[tiab] OR forecast*[tiab] OR prognos*[tiab] OR "risk assess*"[tiab] OR "risk stratif*"[tiab] OR "risk model*"[tiab] | 2600786 |
| 9 | #7 OR #8 | 2935652 |
| 10 | #3 AND #6 AND #9 | 440 |

**Scopus**

| Scopus (date searched: January 1, 2010 to July 14, 2026): | | |
| --- | --- | --- |
|  |  |  |
| 1 | ( TITLE-ABS-KEY (  "artificial intelligence"  OR "machine intelligence" OR "machine learning"  OR "deep learning" OR "data mining"  OR "supervised learning" OR "unsupervised learning"  OR "reinforcement learning" OR "transfer learning"  OR "ensemble learning" OR "ensemble method"  OR "neural network" OR "artificial neural network" OR ANN OR "convolutional neural network" OR CNN OR "recurrent neural network" OR RNN OR transformer*  OR "support vector" OR SVM  OR "random forest" OR "decision tree"  OR "gradient boost*" OR XGBoost OR AdaBoost OR LightGBM OR CatBoost  OR "naive bayes" OR bayesian OR "k-nearest neighbor" OR KNN  OR LASSO  OR classifier* OR "classification algorithm" ) ) | 3934221 |
| 2 | ( TITLE-ABS-KEY (  "surgical site infection" OR SSI OR SSIs  OR "surgical wound infection"  OR "postoperative infection" OR "post-operative infection"  OR "post operative infection"  OR "postoperative wound infection"  OR "postsurgical infection" OR "post-surgical infection"  OR "incisional infection" OR "incision site infection"  OR "operative wound infection"  OR "wound infection" OR "surgical infection"  ) ) | 142466 |
| 3 | ( TITLE-ABS-KEY ( predict* OR forecast* OR prognos* OR "risk assess*" OR "risk stratif*" ) ) | 6636150 |
| 4 | #1 AND #2 AND #3 | 1042 |

**Embase**

| Embase: (date searched: January 1, 2010 to July 14, 2026) | | |
| --- | --- | --- |
|  |  |  |
| 1 | 'machine learning'/exp OR 'machine learning':ti,ab,kw | 771820 |
| 2 | 'artificial intelligence'/exp OR 'artificial intelligence':ti,ab,kw | 221479 |
| 3 | 'deep learning'/exp OR 'deep learning':ti,ab,kw | 152369 |
| 4 | 'data mining'/exp OR 'data mining':ti,ab,kw | 42043 |
| 5 | 'supervised machine learning'/exp OR 'supervised learning':ti,ab,kw OR 'unsupervised learning':ti,ab,kw OR 'reinforcement learning':ti,ab,kw OR 'transfer learning':ti,ab,kw | 43652 |
| 6 | 'artificial neural network'/exp OR 'neural network*':ti,ab,kw OR 'artificial neural network*':ti,ab,kw OR ANN:ti,ab,kw OR 'convolutional neural network*':ti,ab,kw OR CNN:ti,ab,kw OR 'recurrent neural network*':ti,ab,kw OR RNN:ti,ab,kw OR transformer*:ti,ab,kw | 285873 |
| 7 | 'support vector machine'/exp OR 'support vector':ti,ab,kw OR SVM:ti,ab,kw | 78323 |
| 8 | 'random forest'/exp OR 'random forest*':ti,ab,kw | 73391 |
| 9 | 'decision tree'/exp OR 'decision tree*':ti,ab,kw | 38516 |
| 10 | 'bayesian network'/exp OR 'bayesian':ti,ab,kw OR 'naive bayes':ti,ab,kw | 103455 |
| 11 | 'gradient boost*':ti,ab,kw OR adaboost*:ti,ab,kw OR xgboost*:ti,ab,kw  OR lightgbm:ti,ab,kw OR catboost:ti,ab,kw OR 'boosting algorithm*':ti,ab,kw | 33952 |
| 12 | 'ensemble learning':ti,ab,kw OR 'ensemble method*':ti,ab,kw | 7087 |
| 13 | 'k-nearest neighbor*':ti,ab,kw OR KNN:ti,ab,kw | 13580 |
| 14 | LASSO:ti,ab,kw | 36017 |
| 15 | 'classification algorithm*':ti,ab,kw OR classifier*:ti,ab,kw | 75863 |
| 16 | #1 OR #2 OR #3 OR #4 OR #5 OR #6 OR #7 OR #8 OR #9 OR #10 OR #11 OR #12 OR #13 OR#14 OR#15 | 1105036 |
| 17 | 'surgical infection'/exp OR 'surgical infection':ti,ab,kw OR 'surgical site infection*':ti,ab,kw OR 'surgical wound infection*':ti,ab,kw | 75057 |
| 18 | 'wound infection'/exp OR 'wound infection*':ti,ab,kw OR 'operative wound infection*':ti,ab,kw OR 'postoperative wound infection*':ti,ab,kw | 61836 |
| 19 | 'postoperative infection':ti,ab,kw OR 'post-operative infection':ti,ab,kw OR 'post operative infection':ti,ab,kw OR 'postsurgical infection':ti,ab,kw OR 'post-surgical infection':ti,ab,kw OR 'incisional infection*':ti,ab,kw OR 'incision site infection*':ti,ab,kw | 8594 |
| 20 | SSI:ti,ab,kw OR SSIs:ti,ab,kw | 19808 |
| 21 | #17 OR #18 OR #19 OR #20 | 136448 |
| 22 | predict*:ti,ab,kw OR forecast*:ti,ab,kw OR prognos*:ti,ab,kw OR 'risk assess*':ti,ab,kw OR 'risk stratif*':ti,ab,kw | 3892102 |
| 23 | #16 AND #21 AND #22 | 786 |

**Web of Science**

| Web Science Core Collection (date searched: January 1, 2010 to July 14, 2026) | | |
| --- | --- | --- |
|  |  |  |
| 1 | TS=("artificial intelligence" OR "machine intelligence" OR "machine learning" OR "deep learning" OR "data mining" OR "supervised learning" OR "unsupervised learning" OR "reinforcement learning" OR "transfer learning" OR "ensemble learning" OR "ensemble method$" OR "neural network$" OR "artificial neural network$" OR ANN OR "convolutional neural network$" OR CNN OR "recurrent neural network$" OR RNN OR transformer* OR "support vector" OR SVM OR "random forest$" OR "decision tree$" OR "gradient boost*" OR XGBoost OR AdaBoost OR LightGBM OR CatBoost OR bayesian OR "naive bayes" OR "k-nearest neighbor$" OR KNN OR classifier* OR "classification algorithm$") | 1423263 |
| 2 | TS=(surgi* NEAR/2 infect* OR "surgical site infection$" OR SSI OR SSIs OR postoperative NEAR/2 infect* OR "post-operative" NEAR/2 infect* OR "post operative" NEAR/2 infect* OR postsurgical NEAR/2 infect* OR "post-surgical" NEAR/2 infect* OR "incisional infection$" OR "incision site infection$" OR "wound infection$" OR "operative wound infection$") | 42944 |
| 3 | TS=(predict* OR forecast* OR prognos* OR "risk assess*" OR "risk stratif*") | 3165855 |
| 4 | #1 AND #2 AND #3 | 540 |

**CINAHL**

| CINAHL Complete (date searched: January, 2010 to July, 2026): | | |
| --- | --- | --- |
|  |  |  |
| 1 | (MH "Artificial Intelligence+") OR (MH "Machine Learning+") OR (MH "Deep Learning") OR (MH "Neural Networks (Computer)+") OR (MH "Support Vector Machines") OR (MH "Decision Trees+") OR (MH "Bayes Theorem") OR (MH "Data Mining") | 61088 |
| 2 | TI("artificial intelligence" OR "machine learning" OR "deep learning" OR "data mining" OR "neural network*" OR "artificial neural network*" OR ANN OR "convolutional neural network*" OR CNN OR "recurrent neural network*" OR RNN OR transformer* OR "support vector*" OR SVM OR "random forest*" OR "decision tree*" OR "gradient boost*" OR XGBoost OR AdaBoost OR LightGBM OR CatBoost OR "naive bayes" OR bayesian OR "k-nearest neighbor*" OR KNN OR "ensemble learning" OR "ensemble method*" OR "supervised learning" OR "unsupervised learning" OR "reinforcement learning" OR "transfer learning" OR (classifi* AND algorithm*)) | 46576 |
| 3 | AB("artificial intelligence" OR "machine learning" OR "deep learning" OR "data mining" OR "neural network*" OR "artificial neural network*" ANN OR "convolutional neural network*" OR CNN OR "recurrent neural network*" OR RNN OR transformer* OR "support vector*" OR SVM OR "random forest*" OR "decision tree*" OR "gradient boost*" OR XGBoost OR AdaBoost OR LightGBM OR CatBoost OR "naive bayes" OR bayesian OR "k-nearest neighbor*" OR KNN OR "ensemble learning" OR "ensemble method*" OR "supervised learning" OR "unsupervised learning" OR "reinforcement learning" OR "transfer learning" OR (classifi* AND algorithm*) | 86711 |
| 4 | S1 OR S2 OR S3 | 112050 |
| 5 | (MH "Surgical Wound Infection") OR (MH "Wound Infection+") | 13123 |
| 6 | TI("surgical site infection*" OR SSI OR SSIs OR "surgical wound infection*" OR "postoperative wound infection*" OR "postoperative infection*" OR "post-operative infection*" OR "post operative infection*" OR "postsurgical infection*" OR "post-surgical infection*" OR "incisional infection*" OR "incision site infection*" OR "operative wound infection*" OR "wound infection*") | 5003 |
| 7 | AB("surgical site infection*" OR SSI OR SSIs OR "surgical wound infection*" OR "postoperative wound infection*" OR "postoperative infection*" OR "post-operative infection*" OR "post operative infection*" OR "postsurgical infection*" OR "post-surgical infection*" OR "incisional infection*" OR "incision site infection*" OR "operative wound infection*" OR "wound infection*") | 11578 |
| 8 | S5 OR S6 OR 7 | 20494 |
| 9 | (MH "Risk Assessment") OR (MH "Models, Statistical+") OR (MH "Decision Support Techniques+") | 247840 |
| 10 | TI(predict* OR forecast* OR prognos* OR "risk assess*" OR "risk stratif*" OR "risk model*") | 211826 |
| 11 | AB(predict* OR forecast* OR prognos* OR "risk assess*" OR "risk stratif*" OR "risk model*") | 433760 |
| 12 | S9 OR S10 OR S11 | 727159 |
| 13 | S4 AND S8 AND S12 | 131 |

**CNKI**

| CNKI (date searched: January 1, 2010 to July 14, 2026): | | |
| --- | --- | --- |
|  |  |  |
| 1 | ( Subject: "machine learning" OR "machine learning algorithm*" OR "machine learning model*" OR "artificial intelligence" OR "artificial intelligence technology" OR "deep learning" OR "deep learning model*" OR "deep learning method*" OR "deep learning algorithm*" )  AND ( Subject: "postoperative infection*" OR "postoperative infection rate" OR "surgical site infection*" OR "SSI" OR "SSIs" OR "wound" OR "wound infection*" OR "postoperative wound infection*" OR "surgical wound infection*" )  AND ( Subject: "prediction" OR "predictive analysis" OR "prediction model*" OR "analytical prediction" OR "analysis and prediction" OR "predict*" ) | 426 |

2. **Results of initial research**

**PubMed**

| PubMed (date searched: January 1, 2010 to August 9, 2025): | | |
| --- | --- | --- |
|  |  |  |
| 1 | "artificial intelligence"[MeSH Terms] OR "artificial intelligence"[Title/Abstract]  OR "machine intelligence"[Title/Abstract] OR"machine learning"[MeSH Terms] OR "machine learning"[Title/Abstract]  OR"deep learning"[MeSH Terms] OR "deep learning"[Title/Abstract]  OR(classifi*[Title/Abstract] AND algorithm*[Title/Abstract])  OR"Boosting Machine Learning Algorithms"[MeSH Terms]  OR gradient boost*[Title/Abstract] OR boost* algorithm[Title/Abstract] OR adaboost*[Title/Abstract] OR xgboost*[Title/Abstract] OR lightgbm[Title/Abstract] OR catboost[Title/Abstract]  OR"Bayes Theorem"[MeSH Terms] OR bayes*[Title/Abstract]  OR"Support Vector Machine"[MeSH Terms] OR "support vector"[Title/Abstract]  OR"Random Forest"[MeSH Terms] OR random forest*[Title/Abstract]  OR"Decision Trees"[MeSH Terms] OR decision tree*[Title/Abstract] | 504287 |
| 2 | "Surgical Wound Infection"[MeSH Terms]  OR surgical wound infection*[Title/Abstract]  OR postoperative wound infection*[Title/Abstract]  OR post-operative infection*[Title/Abstract]  OR surgical site infection*[Title/Abstract]  OR postoperative infection*[Title/Abstract]  OR incisional infection*[Title/Abstract]  OR "wound infection"[MeSH Terms]  OR wound infection*[Title/Abstract] | 51041 |
| 3 | predict*[Title/Abstract]  OR forecast*[Title/Abstract]  OR screen*[Title/Abstract]  OR monitor*[Title/Abstract]  OR surveillance[Title/Abstract]  OR assess*[Title/Abstract] | 5732186 |
| 4 | #1 AND #2 AND #3 | 365 |

**Scopus**

| Scopus (date searched: January 1, 2010 to August 9, 2025): | | |
| --- | --- | --- |
|  |  |  |
| 1 | (TITLE-ABS-KEY ( "artificial intelligence" OR "machine intelligence" OR "machine learning" OR "deep learning" OR classifi* algorithm* OR gradient boost* OR Adaboost* OR XGBoost* OR Lightgbm OR CatBoost OR “Neural Network” OR "Neural Networks" OR Bayes* OR "Random forest" OR "support vector" OR "Decision Tree" )) | 463554 |
| 2 | TITLE-ABS-KEY ( ( Surg* W/2 Infection* ) OR "surgical site infection" OR "surgical infection" OR ( postoperative W/2 infection* ) OR "postoperative infection" OR incisional infection* OR "incisional infection" OR wound infection* OR "wound infection" ) | 99757 |
| 3 | ( TITLE-ABS-KEY ( predict* OR forecast* OR prognos* OR "risk assess*" OR "risk stratif*" ) ) | 1422101 |
| 4 | #1 AND #2 AND #3 | 100 |

**Embase**

| Embase: (date searched: January 1, 2010 to August 9, 2025) | | |
| --- | --- | --- |
|  |  |  |
| 1 | 'machine learning'/exp OR 'machine learning':ab,ti | 600109 |
| 2 | 'artificial intelligence'/exp OR 'artificial intelligence':ab,ti | 144713 |
| 3 | 'deep learning'/exp OR 'deep learning':ab,ti | 105773 |
| 4 | 'classification algorithm'/exp OR 'classifi* algorithm*':ab,ti | 20724 |
| 5 | 'artificial neural network'/exp OR 'neural network':ab,ti | 158110 |
| 6 | 'boosting machine learning algorithm'/exp OR 'boosting machine learning algorithm':ab,ti | 159 |
| 7 | gradient:ab,ti OR boost*:ab,ti OR adaboost*:ab,ti OR xgboost*:ab,ti OR lightgbm:ab,ti OR catboost:ab,ti | 311313 |
| 8 | 'random forest'/exp OR 'random forest*':ab,ti | 53301 |
| 9 | 'decision tree'/exp OR 'decision tree*':ab,ti | 32147 |
| 10 | 'bayesian network'/exp OR 'bayes*':ab,ti | 88975 |
| 11 | 'support vector machine'/exp OR 'support vector':ab,ti | 60884 |
| 12 | #1 OR #2 OR #3 OR #4 OR #5 OR #6 OR #7 OR #8 OR #9 OR #10 OR #11 | 1049298 |
| 13 | 'surgical infection'/exp OR 'surgical infection':ab,ti | 70743 |
| 14 | 'surgical site infection*':ab,ti OR 'surgical wound infection*':ab,ti OR 'postoperative wound infection*':ab,ti OR 'post-operative wound infection':ab,ti OR 'post-operative wound infection*':ab,ti | 33919 |
| 15 | 'wound infection'/exp OR 'wound infection':ab,ti | 57122 |
| 16 | 'postoperative infection':ab,ti OR 'post-operative infection':ab,ti | 7483 |
| 17 | #13 OR#14 OR#15 OR#16 | 128111 |
| 18 | 'predict*':ab,ti OR 'monitor*':ab,ti OR 'screen*':ab,ti OR 'assess*':ab,ti OR 'surveillance':ab,ti OR 'forecast*':ab,ti | 8521961 |
| 19 | #12 AND #18 AND #19 |  |
| 20 | SSI:ti,ab,kw OR SSIs:ti,ab,kw | 19808 |
| 21 | #17 OR #18 OR #19 OR #20 | 136448 |
| 22 | predict*:ti,ab,kw OR forecast*:ti,ab,kw OR prognos*:ti,ab,kw OR 'risk assess*':ti,ab,kw OR 'risk stratif*':ti,ab,kw | 3892102 |
| 23 | #16 AND #21 AND #22 | 809 |

**Web of Science**

| Web Science Core Collection (date searched: January 1, 2010 to August 9, 2025) | | |
| --- | --- | --- |
|  |  |  |
| 1 | TI=("artificial intelligence" OR "machine intelligence" OR "machine learning" OR "deep learning" OR "classifi* algorithm$" OR classifier OR "Gradient Boost*" OR Adaboost* OR XGBoost* OR LightGBM OR CatBoost OR "Neural Network$" OR Bayes* OR "random forest$" OR"support vector" OR "decision tree$") | 402273 |
| 2 | AB=("artificial intelligence" OR "machine intelligence" OR "machine learning" OR "deep learning" OR "classifi* algorithm$" OR classifier OR "Gradient Boost*" OR Adaboost* OR XGBoost* OR LightGBM OR CatBoost OR "Neural Network$" OR Bayes* OR "random forest$"  OR "support vector" OR "decision tree$") | 867358 |
| 3 | #1 OR #2 | 928640 |
| 4 | TI=(surgi* NEAR/2 infect* OR "surgical site infection$" OR "surgical wound infection$" OR "surgical infection" OR post?operative NEAR/2 infect* OR "post$operative infection" OR "incisional infection$" OR "wound infection$" ) | 7039 |
| 5 | AB=(surgi* NEAR/2 infect* OR "surgical site infection$" OR "surgical wound infection$" OR"surgical infection" OR post?operative NEAR/2 infect* OR "post$operative infection" OR "incisional infection$" OR "wound infection$" ) | 24087 |
| 6 | #4 OR #5 | 26599 |
| 7 | TI=(predict* OR forecast* OR screen* OR assess* OR monitor* OR surveillance) | 1367203 |
| 8 | AB=(predict* OR forecast* OR screen* OR assess* OR monitor* OR surveillance) | 5128499 |
| 9 | #7 OR #8 | 5585206 |
| 10 | #3 AND #6 AND #9 | 211 |
