# Supplementary material for: Explainable Machine Learning Predictive Models for Surgical Site Infections: Scoping Review
Source: J Med Internet Res. 2026 Sep 30;28:e94617. doi: 10.2196/94617 (PMC13626550; doi:10.2196/94617)
Supplement: Supplementary file 2 [file jmir-v28-e94617-s002.docx]

Multimedia Appendix 3: Content Description of Each Feature Category

| **No** | **Feature Category** | **Definition** | **Content Description** |
| --- | --- | --- | --- |
| 1 | Individual Characteristics | Baseline sociodemographic and intrinsic patient attributes describing a patient’s fundamental identity and social structure | (1) Age;  (2) Gender;  (3) Race/Ethnicity;  (4) Educational Level;  (5) Marriage Status |
| 2 | Health and Lifestyle | Patient-level physiological status and modifiable health-related behaviors or composite health indicators, including anthropometric measures, functional and perioperative risk scores, as well as lifestyle exposures | (1) Height/Weight/BMI;  (2) Any Health Assessment Indicator (e.g., ASA Classification, Functional Health Status, Body Surface Area (BSA));  (3) Tobacco and Alcohol Use |
| 3 | Comorbidity and Treatment | Presence of pre-existing systemic diseases across organ systems and their corresponding therapeutic management prior to the index admission or surgical intervention | Any Comorbidity and Their Corresponding Treatment (e.g., Endocrine Disease, Heart Illnesses, Digestive System Disease, Kidney Disease, Respiratory System Disease, Cancer, Cerebrovascular Diseases, Nervous System Disease, Immune System Disease, Orthopedic Diseases, and others) |
| 4 | Surgical Setting | Institutional, organizational, and contextual characteristics of the surgical care environment | (1) Preoperative Diagnosis (Surgical Indication);  (2) Any Information about Local Health Authority;  (3) Any information about Hospital;  (4) Any Device or Equipment used in this Surgery;  (5) Surgical Team Personnel;  (6) Surgical Venue |
| 5 | Preoperative Preparation | All preventive and optimization interventions performed prior to surgery to reduce perioperative risk and improve surgical outcomes | (1) Cardiovascular Preparation;  (2) Gastrointestinal Preparation;  (3) Respiratory Tract Preparation;  (4) Urinary Catheterization;  (5) Skin Preparation;  (6) Blood Prodcut Preparation;  (7) Antibiotics Preparation |
| 6 | Surgical Technique | Core procedural strategy and operative approach used to perform surgery | (1) Surgical Specialty; (2) Surgical Approach (e.g., Open/Minimally Invasive/Robot);  (3) Surgical Type (Elective/Emergency);  (4) Anesthesia |
| 7 | Surgical Process Details | Detailed intraoperative procedural characteristics describing technical execution and operative complexity | (1) Surgical Time; (2) Intraoperative Finding;  (3) Anatomic Localization; (4) Suture Pattern;  (5) Anastomosis; (6) Knot-Tying;  (7) Devices/Supplies/Drugs/Blood Products Used;  (8) Non-Conventional Maneuver;  (9) Determination of Surgical Margins;  (10) Incision Classification/Length/Size/Depth/Angle/Number |

| **No** | **Feature Category** | **Definition** | **Content Description** |
| --- | --- | --- | --- |
| 8 | Admission Management | Administrative and clinical processes during patient admission and hospitalization flow | (1) Length of Admission;  (2) Inpatient/Outpatient;  (3) Transfer Origin;  (4) ICU Use |
| 9 | Vital Signs | Standard physiological parameters reflecting immediate systemic status and hemodynamic stability | (1) Body Temperature;  (2) Heart Rate;  (3) Pulse;  (4) Respiratory Rate;  (5) Blood Pressure |
| 10 | Inflammatory Markers | Laboratory biomarkers reflecting systemic immune activation, inflammatory response, and immune cell dynamics | (1) White Blood Cell (WBC);  (2) Neutrophil percentage (%);  (3) Lymphocyte percentage (%);  (4) Lymphocytes Count;  (5) Monocytes Percentage (%);  (6) Monocytes Count;  (7) Eosinophils Count;  (8) Eosinophils Percentage (%);  (9) Basophils Percentage (%);  (10) lymphocyte to monocyte ratio (LMR);  (11) Platelets Count (PLT);  (12) Erythrocyte Sedimentation Rate (ESR)  (13) Globulin;  (14) C-reactive protein;  (15) Procalcitonin (PCT);  (16) Tumor Necrosis Factor-alpha (TNF-α);  (17) Interleukin-6 (IL-6)  (18) Systemic Immune-Inflammation Index (SII);  (19) Aggregate Index of Systemic Inflammation (AISI);  (20) CRP-albumin-lymphocyte (CALLY) |
| 11 | Oxygen-Carrying and Anemia Markers | Hematological indicators of oxygen transport capacity and erythrocyte status | (1) Hemoglobin (Hb);  (2) Red Blood Cell (RBC);  (3) Hematocrit  (4) Mean Corpuscular Hemoglobin Concentration; |
| 12 | Coagulation Function Markers | Hemostatic and coagulation-related laboratory parameters reflecting clot formation, fibrinolysis, and anticoagulant balance | (1) Fibrinogen;  (2) Prothrombin Time (PT);  (3) Partial Thromboplastin Time (PTT);  (4) Activated Partial Thromboplastin Time (APTT);  (5) Thrombin Time;  (6) D-Dimer;  (7) International Normalized Ratio (INR) |
| 13 | Nutritional and Metabolism Markers | Biochemical indicators of nutritional status and systemic metabolic regulation | (1) Albumin; (2) Prealbumin;  (3) Total Protein; (4) Glucose Examinations indicators;  (5) Lipid-Related Examination indicators |
| 14 | Hepatobiliary Function Markers | Biochemical indicators of hepatic metabolism, biliary excretion, and hepatocellular integrity | (1) Bilirubin;  (2) Direct Bilirubin;  (3) Indirect Bilirubin;  (4) Total Bile Acid;  (5) Alanine Transaminase/Alanine Aminotransferase (ALT);  (6) Aspartate Aminotransferase/Glutamate Oxaloacetate Transaminase/ Aspartate Transaminase (AST);  (7) Alkaline Phosphatase |
| 15 | Renal Function Markers | Laboratory measures of renal filtration, excretory function, and nitrogen metabolism | (1) Blood Urea Nitrogen (BUN);  (2) Creatinine;  (3) Uric Acid;  (4) estimated Glomerular Filtration Rate (eGFR) |
| 16 | Electrolyte and Mineral Markers | Serum concentrations of essential electrolytes and minerals required for physiological homeostasis | (1) Potassium;  (2) Sodium;  (3) Calcium;  (4) Phosphorus;  (5) Magnesium |
| 17 | Acid-Base and Respiratory Status Markers | Arterial or venous blood gas parameters reflecting acid-base equilibrium and pulmonary gas exchange efficiency, assessed through blood gas analysis. | Blood Gas Analysis-Related Examination |
| 18 | Microbiological Findings | Microbiological test results identifying infectious pathogens and fungal organisms through culture-based or laboratory diagnostic methods. | (1) Bacteria Culture;  (2) Fungi Culture |
| 19 | Recovery Status | Postoperative or post-treatment clinical recovery indicators reflecting tissue healing, symptom resolution, drainage condition, and use or removal of indwelling medical devices. | (1) Wound Recovery;  (2) Symptoms;  (3) Drainage Status;  (4) Indwelling Devices Utilization |
| 20 | Clinical Intervention | Therapeutic and diagnostic interventions administered during outpatient or inpatient care | (1) Postoperative Pain Relief;  (2) Postoperative Antibiotics Administration;  (3) Postoperative Diagnosis;  (4) Postoperative Examination |
| 21 | Administrative Code | Standardized clinical classification codes used for documentation, diagnosis, procedure identification, and healthcare service reporting | Any Diagnostic Code and Medical Service Code |
