# Supplementary material for: Explainable Machine Learning Predictive Models for Surgical Site Infections: Scoping Review
Source: J Med Internet Res. 2026 Sep 30;28:e94617. doi: 10.2196/94617 (PMC13626550; doi:10.2196/94617)
Supplement: Supplementary file 3 [file jmir-v28-e94617-s003.docx]

**Multimedia Appendix 4:** Assessment of the quality, risk of bias and applicability of included models (n=72)

| **Author, Year** | **Best-performing Model** | **Model development** | | | | | | | | | **Model evaluation** | | | | | | | | |
| --- | --- | --- | --- | --- | --- | --- | --- | --- | --- | --- | --- | --- | --- | --- | --- | --- | --- | --- | --- |
|  |  | **Quality Concern** | | | | | **Applicability Concern** | | | | **Risk of Bias** | | | | | **Applicability Concern** | | | |
|  |  | **participant** | **predictor** | **outcome** | **analysis** | **overall** | **participant** | **predictor** | **outcome** | **overall** | **participant** | **predictor** | **outcome** | **analysis** | **overall** | **participant** | **predictor** | **outcome** | **overall** |
| **Mamlook et al, 2023** | DNN ^a^ | high | unclear | unclear | high | high | low | low | unclear | unclear | high | unclear | unclear | high | high | low | low | unclear | unclear |
| **Ohno et al 2022** | GBT and NN Ensemble Model | high | unclear | unclear | high | high | low | low | low | low | NA | NA | NA | NA | NA | low | low | low | low |
| **Orfanoudaki et al. 2022** | XGBoost ^b^ | high | unclear | unclear | low | high | low | low | low | low | high | unclear | unclear | unclear | high | low | low | low | low |
| **Petrosyan et al,2021** | RF ^c^ using ICD-10 | high | unclear | high | high | high | low | low | low | low | high | unclear | high | high | high | low | low | low | low |
|  | RF using ICD-9-CA | high | unclear | high | high | high | low | low | low | low | high | unclear | high | high | high | low | low | low | low |
|  | RF using Physician Procedure Claims Codes | high | unclear | high | high | high | low | low | low | low | high | unclear | high | high | high | low | low | low | low |
| **Piebpien**  **et al,2024** | NB ^d^ | high | unclear | high | high | high | low | low | low | low | high | unclear | high | high | high | low | low | low | low |
| **Rennert-May et al，2022** | Regularized LR ^e^ | high | high | high | high | high | low | low | low | low | high | high | high | high | high | low | low | low | low |
| **Ruan et al，2022** | Multimodal GRU-D (dynamic variables) | high | unclear | high | high | high | high | low | low | high | high | unclear | high | high | high | high | low | low | high |
|  | Single layer GRU-D (dynamic variables) | high | unclear | high | high | high | high | low | low | high | high | unclear | high | high | high | high | low | low | high |
|  | Single layer GRU-D (dynamic and static variables) | high | unclear | high | high | high | high | low | low | high | high | unclear | high | high | high | high | low | low | high |
| **Salimy et al，2025** | HGBT ^f^ | high | unclear | unclear | high | high | low | low | low | low | high | unclear | unclear | high | high | low | low | low | low |
| **Van et al,2014** | Text SVM ^g^ | high | high | unclear | high | high | low | low | unclear | unclear | high | high | unclear | high | high | low | low | unclear | unclear |
| **Walczak et al,2019** | ANN ^k^ | high | high | unclear | high | high | low | low | unclear | unclear | high | unclear | unclear | unclear | high | low | low | unclear | unclear |
| **Wang et al,2021** | NB | high | unclear | unclear | high | high | low | low | low | low | high | unclear | unclear | high | high | low | low | low | low |
| **Wang et al,2025** | XGBoost | high | unclear | unclear | high | high | low | low | low | low | high | unclear | unclear | high | high | low | low | low | low |
| **Weller et al,2018** | LASSO Regression | high | unclear | high | high | high | low | low | low | low | high | unclear | high | high | high | low | low | low | low |
| **Xiong et al,2022** | AdaBoost Classification Trees model | high | unclear | high | high | high | low | low | low | low | high | unclear | high | high | high | low | low | low | low |
| **Yang et al，2024** | LASSO Logistic Regression | low | unclear | unclear | low | unclear | low | high | low | high | low | unclear | unclear | high | unclear | low | high | low | high |
| **Yu et al,2014** | DT ^l^ | high | unclear | unclear | high | high | low | low | unclear | unclear | high | unclear | unclear | high | high | low | low | unclear | unclear |
| **Zhang et al,2024** | NB | high | unclear | unclear | high | high | low | low | low | low | high | unclear | unclear | high | high | low | low | low | low |
| **Zhu et al,2021** | LASSO ^m^ regression (superficial) | high | unclear | unclear | high | high | low | low | low | low | high | high | unclear | high | high | low | low | low | low |
|  | LASSO regression (organ/space) | high | unclear | unclear | high | high | low | low | low | low | high | high | unclear | high | high | low | low | low | low |
|  | LASSO regression (composite) | high | unclear | unclear | high | high | low | low | low | low | high | high | unclear | high | high | low | low | low | low |
| **Chuyang et al，2017** | An enhanced regression model | low | unclear | low | unclear | unclear | low | low | low | low | low | unclear | low | high | high | low | low | low | low |
|  |  |  |  |  |  |  |  |  |  |  |  |  |  |  |  |  |  |  |  |
| **Alexander et al, 2021** | DNN for superficial SSI | high | unclear | unclear | high | high | low | low | unclear | unclear | high | high | unclear | high | high | low | low | unclear | unclear |
|  | DNN for deep SSI | high | unclear | unclear | high | high | low | low | unclear | unclear | high | unclear | unclear | high | high | low | low | unclear | unclear |
|  | DNN for organ/space SSI | high | unclear | unclear | high | high | low | low | unclear | unclear | high | unclear | unclear | high | high | low | low | unclear | unclear |
| **Bonde et al, 2024** | DNN for superficial SSI | high | high | unclear | unclear | high | low | low | high | high | high | high | unclear | high | high | low | low | high | high |
|  | DNN for deep SSI | high | high | unclear | unclear | high | low | low | high | high | high | high | unclear | high | high | low | low | high | high |
|  | DNN for organ/space SSI | high | high | unclear | unclear | high | low | low | high | high | high | high | unclear | high | high | low | low | high | high |
| **Grass et al. 2021** | BPMI ^n^ | high | unclear | unclear | high | high | low | low | low | low | high | unclear | unclear | high | high | low | low | low | low |
| **Colborn et al,2023** | Regularized logistic model | high | unclear | unclear | high | high | low | low | high | high | high | unclear | unclear | high | high | low | low | high | high |
| **Chen et al,2020** | The Self-Attention Network | high | low | unclear | unclear | high | low | low | low | low | high | high | unclear | high | high | low | low | low | low |
| **Kiser et al,2024** | LSTM ^o^ | high | high | high | high | high | low | low | low | low | high | high | high | high | high | low | low | low | low |
| **Colborn et al,2018** | LASSO Regression | high | unclear | unclear | high | high | low | low | unclear | unclear | high | unclear | unclear | high | high | low | low | unclear | unclear |
| **Julien et al,2022** | RF | low | low | high | high | high | low | low | high | high | low | low | high | high | high | low | low | high | high |
| **Chen et al,2023** | NN ^p^ | high | unclear | high | high | high | low | low | low | low | high | unclear | high | high | high | low | low | low | low |
| **Chen et al,2024** | RF | high | high | unclear | high | high | low | low | low | low | high | unclear | unclear | high | high | low | low | low | low |
| **Kilic et al,2021** | XGBoost | high | unclear | unclear | high | high | low | low | low | low | high | unclear | unclear | high | high | low | low | low | low |
| **Golinelli et al,2025** | XGBoost | high | unclear | unclear | high | high | low | low | low | low | high | unclear | unclear | high | high | low | low | low | low |
| **Isbell et al,2021** | Regularized Bayesian Multilevel Logistic Regression | high | unclear | unclear | high | high | low | low | low | low | high | unclear | unclear | high | high | low | low | low | low |
| **Zhuang et al,2024** | LASSO Logistic Regression | high | unclear | unclear | high | high | low | low | low | low | high | unclear | unclear | high | high | low | low | low | low |
| **McLean et al,2024** | LASSO Logistic Regression | high | unclear | unclear | high | high | low | low | low | low | high | unclear | unclear | unclear | high | low | low | low | low |
| **Wei et al,2020** | Regularized Bayesian plus LR | high | high | high | high | high | low | low | low | low | high | high | high | high | high | low | low | low | low |
| **Betts et al,2019** | XGBoost | high | high | high | high | high | high | low | high | high | high | high | unclear | high | high | high | low | high | high |
| **Erik et al,2022** | LASSO Regression | high | high | high | high | high | high | low | low | high | high | high | high | high | high | high | low | low | high |
| **Cui et al,2025** | GBM ^q^ | high | high | low | high | high | low | low | low | low | high | high | low | high | high | low | low | low | low |
| **FloreshighBalado et al,2023** | XGBoost | high | high | high | high | high | low | low | high | high | high | high | high | high | high | low | low | high | high |
| **Hopskin et al,2020** | DNN | high | high | unclear | high | high | low | low | high | high | high | high | unclear | high | high | low | low | high | high |
| **Jurij et al,2025** | XGBoost | high | low | high | high | high | low | low | high | high | high | low | high | high | high | low | low | high | high |
| **Li et al,2024** | RF | high | high | high | high | high | low | low | low | low | high | high | high | high | high | low | low | low | low |
| **Liu et al,2022** | XGB | high | high | low | unclear | high | low | low | low | low | high | high | high | unclear | high | low | low | low | low |
| **Ingwon et al,2022** | ANN | high | high | high | unclear | high | low | low | unclear | unclear | high | high | high | high | high | low | low | unclear | unclear |
| **Ying et al,2023** | ET ^r^ | high | high | high | high | high | low | low | low | low | high | high | high | high | high | low | low | low | low |
| **Liang et al,2025** | RF | high | high | high | high | high | low | low | low | low | high | high | high | high | high | low | low | low | low |
| **Liao et al,2018** | ANN | high | low | high | high | high | low | low | low | low | high | low | high | high | high | low | low | low | low |
| **Hu et al,2024** | DT | low | low | high | high | high | low | low | low | low | low | high | high | high | high | low | low | low | low |
| **Cheng et al,2024** | Meta-Lasso with class 1 | high | unclear | high | high | high | low | low | unclear | unclear | high | high | high | high | high | low | low | unclear | unclear |
|  | Meta-Lasso with class 2 | high | unclear | high | high | high | low | low | unclear | unclear | high | high | high | high | high | low | low | unclear | unclear |
| **Gutierrez Naranjo et al,2024** | NN | high | high | high | high | high | low | low | low | low | high | high | high | high | high | low | low | low | low |
|  | Boosted generalized linear model | high | high | high | high | high | low | low | low | low | high | high | high | high | high | low | low | low | low |
|  | NB | high | high | high | high | high | low | low | low | low | high | high | high | high | high | low | low | low | low |
|  | Penalized discriminant analysis | high | high | high | high | high | low | low | low | low | high | high | high | high | high | low | low | low | low |
| **Wang et al,2025** | LASSO logistic regression | high | high | high | high | high | low | low | low | low | high | high | high | high | high | low | low | low | low |
|  |  |  |  |  |  |  |  |  |  |  |  |  |  |  |  |  |  |  |  |
| **Xu et al,2020** | RF using class 1 | high | high | unclear | unclear | high | high | low | unclear | high | high | high | unclear | high | high | high | low | unclear | high |
|  | RF using class 2 | high | high | unclear | unclear | high | high | low | unclear | high | high | high | unclear | high | high | high | low | unclear | high |
| **Song et al,2025** | RF | high | low | high | high | high | low | low | unclear | unclear | high | high | unclear | high | high | low | low | unclear | unclear |
| **Long et al,2025** | LASSO  LR | high | high | high | high | high | low | low | low | low | high | high | high | high | high | low | low | low | low |
| **Choi et al,2024** | RF | high | unclear | high | unclear | high | low | unclear | high | high | high | high | high | unclear | high | low | unclear | high | high |
| **Pang et al,2025** | SVM | high | high | high | high | high | low | low | low | low | high | high | high | high | high | low | low | low | low |
| **Janneke et al,2023** | DT | high | high | high | high | high | low | low | low | low | high | high | high | unclear | high | low | low | low | low |
| **Agostinho et al,2025** | NB | low | unclear | low | unclear | unclear | low | low | low | low | low | unclear | low | high | high | low | low | low | low |
|  | DNN | low | unclear | low | unclear | unclear | low | low | low | low | low | unclear | low | high | high | low | low | low | low |
| **Phuyal et al,2026** | XGBoost | high | unclear | unclear | unclear | high | low | low | low | low | high | unclear | high | high | high | low | low | low | low |
| **Nie et al,2026** | RF | high | high | high | high | high | low | low | low | low | high | high | high | high | high | low | low | low | low |
| **Jha et al,2026** | SVM | low | low | unclear | high | high | low | low | unclear | unclear | low | low | unclear | high | high | low | low | unclear | unclear |
| **Ghabisha et al,2026** | XGBoost | high | unclear | low | high | high | low | low | low | low | high | unclear | low | unclear | high | low | low | low | low |
| **Cao et al,2026** | Stacking models  (Pre-LIFT) | high | unclear | high | unclear | high | low | unclear | low | unclear | high | unclear | high | high | high | low | unclear | low | unclear |
|  | Stacking models  (Post-LIFT) | high | unclear | high | unclear | high | low | unclear | low | unclear | high | unclear | high | high | high | low | unclear | low | unclear |
| **Li et al,2025** | DT | high | unclear | high | unclear | high | low | low | low | low | high | unclear | high | unclear | high | low | low | low | low |
| **De et al,2025** | RF | unclear | unclear | high | high | high | low | low | low | low | unclear | unclear | high | high | high | low | low | low | low |
| **Michael**  **et al,2025** | XGBoost  (Maximum performance models) | unclear | low | unclear | unclear | unclear | unclear | unclear | low | unclear | unclear | low | unclear | low | unclear | unclear | unclear | low | unclear |
|  | XGBoost  (Facility-agnostic models) | unclear | low | unclear | unclear | unclear | unclear | unclear | low | unclear | unclear | low | unclear | low | unclear | unclear | unclear | low | unclear |
| **Celik et al,2025** | XGBoost | high | unclear | high | unclear | high | low | low | low | low | high | unclear | high | unclear | high | low | low | low | low |
| **Bangash et al,2025** | Stacking ensemble model | low | unclear | unclear | low | unclear | low | low | low | low | low | unclear | unclear | high | high | low | low | low | low |
| **Perkins,2024** | LR | high | high | high | high | high | high | low | low | high | high | high | high | high | high | high | low | low | high |
| **Orlandi et al,2022** | LASSO regression | high | high | unclear | unclear | high | low | low | low | low | high | high | unclear | high | high | low | low | low | low |
| **Fijacko et al,2019** | LASSO regression | high | unclear | unclear | high | high | low | high | unclear | high | high | unclear | unclear | high | high | low | high | unclear | high |
| **Gowd et al,2019** | Logistic regression | high | unclear | unclear | high | high | low | low | low | low | high | unclear | unclear | high | high | low | low | low | low |
| **Rahimi et al,2025** | RF | high | unclear | high | high | high | low | low | low | low | high | unclear | high | high | high | low | low | low | low |
|  | DT | high | unclear | high | high | high | low | low | low | low | high | unclear | high | high | high | low | low | low | low |
|  | K-NN | high | unclear | high | high | high | low | low | low | low | high | unclear | high | high | high | low | low | low | low |
|  | XGBoost | high | unclear | high | high | high | low | low | low | low | high | unclear | high | high | high | low | low | low | low |
| **Ying et al,2026** | LightGBM | high | unclear | unclear | high | high | low | unclear | low | unclear | high | unclear | unclear | high | high | low | unclear | low | unclear |
| **Zhang et al,2025** | GBM | high | unclear | unclear | high | high | low | low | low | low | high | unclear | unclear | high | high | low | low | low | low |
| **Zhou et al,2025** | RF | high | unclear | low | high | high | low | low | low | low | high | unclear | low | high | high | low | low | low | low |
| **Li et al,2025** | DT | high | unclear | unclear | high | high | low | low | low | low | high | unclear | unclear | high | high | low | low | low | low |

^a^ DNN: Deep Neural Network

^b^ XGBoost: Extreme Gradient Boosting

^c^ RF: Random Forest

^d^ NB: Native Bayes

^e^ LR: logistic regression

^f^ HBGT: Histogram-Based Gradient Boosting

^g^ SVM: Support Vector Machine

^h^ ANN: Artificial Neural Network

^l^ DT: Decision Tree

^m^ LASSO: Least Absolute Shrinkage and Selection Operator

^n^ BPMI: Bayesian-Probit Regression Model with Multiple-Imputation

^o^ LSTM: Long Short-Term Memory

^p^ NN: Neural Network

^q^ GMB: Gradient Boosting Machine

r ET: Extra Trees Classifier
